# Conveyor-mode electron shuttling through a T-junction in Si/SiGe

**DOI:** 10.1038/s41467-026-78805-1

**Published:** 2026-11-05

**Authors:** Max Beer, Ran Xue, Lennart Deda, Stefan Trellenkamp, Jhih-Sian Tu, Paul Surrey, Inga Seidler, Hendrik Bluhm, Lars R. Schreiber

**Affiliations:** 1 JARA-FIT Institute for Quantum Information, Forschungszentrum Jülich GmbH and RWTH Aachen University, Aachen, Germany; 2 ARQUE Systems GmbH, Aachen, Germany; 3 Helmholtz Nano Facility (HNF), Forschungszentrum Jülich, Jülich, Germany

**Keywords:** Electronic devices, Qubits, Information theory and computation, Quantum dots

## Abstract

Conveyor-mode shuttling in gated Si/SiGe devices enables adiabatic transfer of single electrons, electron patterns and spin qubits confined in quantum dots across several microns with a scalable number of signal lines. To realize their full potential, linear shuttle lanes must connect into a two-dimensional grid with controllable routing. We introduce a T-junction device linking two independently driven shuttle lanes. Electron routing across the junction requires no extra control lines beyond the four channels per conveyor belt. We measure an inter-lane charge transfer fidelity of $F={100.0000000}_{-9\times 1{0}^{-7}}^{+0}\,\%$ at an instantaneous electron velocity of 270 mm s^−1^. The filling of 54 quantum dots is controlled by simple atomic pulses, allowing us to swap electron patterns, laying the groundwork for a native spin-qubit SWAP gate. This T-junction establishes a path towards scalable, two-dimensional quantum computing architectures with flexible spin qubit routing for quantum error correction.

## Abstract (web summary)

Conveyor-mode electron shuttling offers a scalable route to connecting spin qubits, but extension to two-dimensional architectures requires reliable routing between shuttle lanes. Here, the authors demonstrate a silicon T-junction enabling near-unity fidelity transfer of single electrons between conveyor mode channels.

## Introduction

Electron and hole spin quantum computing in electrostatically defined SiGe quantum dots (QDs) has progressed considerably^1,2^: Single- and two-qubit gate fidelities exceeding the error correction threshold^3–6^ as well as scaling of multi-qubit devices up to twelve qubits^7^ have been demonstrated. These scaling efforts manifest as increasingly large (bi-) linear^7–10^ and dense, two-dimensional QD arrays^11–13^. Such dense architectures as well as large linear arrays suffer from limited scalability due to wiring bottlenecks and difficulties in implementing the long-range qubit coupling required^14,15^, while two-dimensional qubit topologies with millions of physical qubits are needed for the implementation of many efficient quantum error correction algorithms^16,17^.

To solve these challenges, qubit shuttling has been investigated intensely. The two dominant methods of qubit shuttling in QDs are bucket-brigade^18–21^ and conveyor-mode^21–25^ shuttling. The latter features higher spin shuttle fidelity and has been demonstrated as the more scalable solution^21,23,24,26,27^ as it demands only a small and constant number of control lines and signals independent of the shuttling distance^28–30^. While conveyor-mode shuttling has already been deployed coupling stationary qubits^31^, the required extension into two dimensions has not been demonstrated so far. Shuttle junctions and qubit routing pose a challenge even for other, arguably more mature qubit platforms such as ion-qubits, for which linear shuttling has been demonstrated much earlier^32–34^. Surface-code quantum error correction for spin-qubits requires thousands of physical qubits to encode one logical qubit and thus millions of single electron spins need to be positioned and controlled. This demands a high fidelity of controlling these electrons, while control signals must be simple to co-integrate signal generation and routing on the chip^15,29^.

Here we demonstrate a key building block, a two-dimensional intersection of orthogonal one-dimensional conveyor-belt shuttle lanes (also called QuBus). This T-junction consists of 54 QDs and enables flexible routing of single electrons. Despite its complex gate design, all routing and filling of all QDs is controlled by only eight simple voltage signals (called atomic pulses). We observe single electron transport across the 10 μm × 5 μm device with an inter-lane charge transfer fidelity of $F={100.0000000}_{-9\times 1{0}^{-7}}^{+0}\,\%$ (See methods section on notation) at an instantaneous electron velocity of 270 mm s^−1^. Already this velocity paves the way to spin coherent shuttling provided the hyperfine interaction is sufficiently low employing isotope purification and the valley splitting energy is sufficiently large^26^. The latter is subject of current research^35–39^ and sufficiently large valley-splitting has been achieved across a multi quantum dot device with an optimized Si/SiGe heterostructure^2^. We demonstrate the first step towards a spin-SWAP gate native to our device topology by permuting initialized QD fillings encoding electron patterns^23^.

## Results

### T-junction device and atomic pulses

**Fig. 1:**
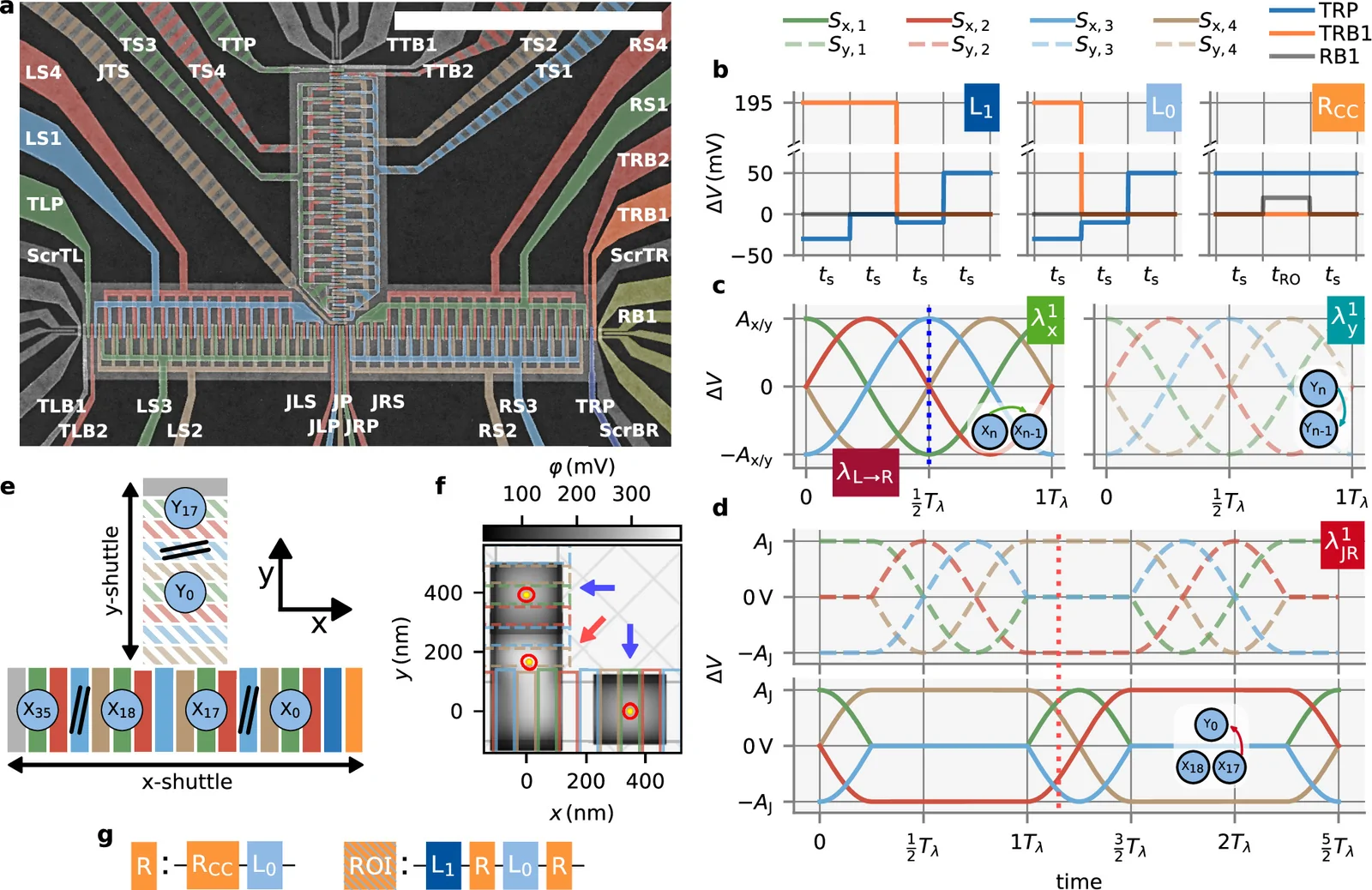
T-junction device and atomic pulses. **a** False color scanning electron micrograph of the surface of a device nominally identical to the device measured, including gate labels. Gate color coding is consistent between all panels and indicates gates comprising gate sets S_x,*i*_ and S_y,*i*_ (see methods). Gates defining the sensor used for electron charge readout are shaded yellow. The scale bar is 5 μm. **b** Atomic pulses for charge initialization and readout. The indicated voltages are typical values. Typical values are *t*_S_ ≈ 10 ms and *t*_RO_ ≈ 100 ms. **c** Horizontal and vertical charge shuttle atomic pulses. The first half of ${\lambda }_{x}^{1}$, up to the dashed line, describes *λ*_L→R_. **d** Junction transfer atomic pulse. Pulses for finger gates in x- and y-shuttle are shown on the bottom and top, respectively. Note that the x- and y-shuttle are not operated simultaneously. Simulated electron wave function for the voltage configuration indicated by the red dashed line is shown in (**f**). **e** Nominal dot positions in the idle state. In the readout state (see methods), all QD X_*n*_ are shifted two gates towards the right. Periodic continuation of more QDs are omitted but indicated with (//) symbols. **f** Simulated potentials *φ* and electron ground state probability densities (yellow-red circular shapes) at three positions in the device corresponding to three points in time. Claviature gates are shown as outlines and screening gates are indicated as a light gray grid. Blue arrows: Electron states in QD X_16_ and QD Y_0_ in the idle state. Red arrow: Electron during inter-lane junction transfer. 95 %, 68 %, 1 % probability density levels shown in yellow, orange and red, respectively. (time-dependence of simulations in Supplementary Movies 1 and 2) **g** Commonly used composite pulses. R: destructive readout pulse. ROI: readout and initialization calibration pulse.

The T-junction is composed of two individual QuBus shuttle lanes, labeled x-shuttle and y-shuttle, joined at a 90^∘^ angle with a single electron transistor (SET) formed at the exterior-facing end of each lane (Fig. 1a). Electrostatic gates used in the operation of the device are labeled in Fig. 1a. The large number of gates and device terminals allows for more flexibility and fine control over the electron confinement potential than required to operate the device in this work. For this reason, most gates are grouped into connected gate sets S_*j*,*i*_ (*j* ∈ {x, y}, *i* ∈ {1, 2, 3, 4}) as indicated by color in Fig. 1a (see methods for details) with the same voltage applied to each gate in a set, significantly reducing the effective number of terminals required to operate the T-junction.

The voltage applied to gate *g* is composed of a static voltage *V*_*g*,0_ and an optional voltage pulse *ΔV*_*g*_*(t)*. We set *V*_*g*,0_ = 330 mV for gates belonging to these sets. We set the gates terminating the shuttle lanes to the left and top, TLB1 and TTB1, to *V*_*g*,0_ = 0 V to define a sharp shuttle lane end.

A pulse *P* is defined as the set of time-dependent voltage sequences simultaneously applied as *ΔV*_*g*_(*t*) individually specified for one or more gates *g*. The voltage applied to gates not specified in the definition of *P* remains constant during the duration of the pulse. The time reverse and *n* consecutive repetitions of *P* are denoted as *P*^−1^ and *P* ^*n*^, respectively. Pulses can be composed of other pulses by applying these immediately after each other. We refer to a pulse that is not composed of other pulses as an atomic pulse, of which we define eight (1 and 0 electron loading L_1_, L_0_ (Fig. 1b); charge readout R_CC_ (Fig. 1b) and preparation $\lambda_{{\mathrm{L}}\rightarrow{\mathrm{R}}}$ (Fig. 1c); linear charge shuttling ${\lambda }_{{{{\rm{x}}}}}^{1}$, ${\lambda }_{{{{\rm{y}}}}}^{1}$ (Fig. 1c); T-junction transfers ${\lambda }_{{{{\rm{JR}}}}}^{1}$, ${\lambda }_{{{{\rm{JL}}}}}^{1}$ (Fig. 1d, Supplementary Fig. 2)), each achieving a specific function. In total, only eleven voltage channels are required to apply all atomic pulses. Despite the device complexity, these eight atomic pulses are sufficient to operate the entire device.

Typical for the conveyor-belt shuttling approach^22,23,28,29^, we define the idle state of the device forming QDs under every fourth gate in the x- and y-shuttle lane, here in total 54 - labeled QD X_*n*_ (*n* ∈ [0, 35]) and QD Y_*m*_ (*m* ∈ [0, 17]) (Fig. 1e). In this idle configuration, the QD minima in the x-shuttle are centered under the S_x,1_ gate set and in the y-shuttle under the S_y,1_ gate set (Fig. 1e). Proximal single-electron charge detection is achieved by measuring the current across the right SET modified by the capacitive coupling of the charge present in QD X_0_ to the chemical potential of the SET. In order to keep the number of signal channels as low as possible to simplify control, we only use the right SET in this work. To increase this capacitive coupling, the readout state is defined placing the QD minima in the x-shuttle under TRP and the S_x,3_ gate set, in closer proximity to the SET at the right end of the x-shuttle. If not otherwise stated, atomic pulses start at and return to the idle state. While conveyor-mode shuttling continuously moves the QDs, we reassign the QD labels after each atomic pulse to match the initial QD positions. A simulation of the electron wavefunction when confined in the QDs is shown in Fig. 1f (see Supplementary Note I for further information).

Starting and ending in the readout state, we calibrate the charge initialization atomic pulses (Fig. 1b) from charge stability measurements to initialize the charge occupancy of QD X_0_ to either one electron (L_1_) or zero (L_0_), by transferring electrons between QD X_0_ and a reservoir formed at the SET at the right end of the x-shuttle, similar to ref. ^23^. The charge occupancy of QD X_0_ in the readout state is determined from the current across the right SET, which we tune to its optimal sensitivity by applying the atomic pulse R_CC_. To reset the QD occupancy for future readout, R_CC_ is always followed by unloading QD X_0_, defining the destructive readout pulse R.

Forward conveyor-belt shuttling in the x-(y-)shuttle lane means a continuous transfer of a QD positioned at QD X_*n*_ to QD X_*n*−1_ (QD Y_*m*_ to QD Y_*m*−1_). To facilitate forward conveyor-belt shuttling, we pulse shuttle gates in the x- and y-shuttle according to

1 $$\Delta {V}_{g\,\in\,{{{{\rm{S}}}}}_{j,i}}\left(t\right)={A}_{j}\sin \left(-\dfrac{2\pi t}{{T}_{\lambda }}+i\cdot \dfrac{\pi }{2}\right)$$

with *i* ∈ {1, 2, 3, 4} and *j* ∈ {x, y} where *T*_*λ*_ is the shuttle period and *A*_x_, *A*_y_ are the drive amplitudes for the x- and y-shuttle, respectively. The voltage pulses applied to S_x,3_ are also applied to TRP. A single period *T*_*λ*_ of this sequence, applied to either the x- or y-shuttle, defines atomic pulses ${\lambda }_{{{{\rm{x}}}}}^{1}$ or ${\lambda }_{{{{\rm{y}}}}}^{1}$ (Fig. 1c), continuously moving a QD a distance of four gates, i.e. *λ* = 280 nm with a nominal instantaneous shuttle velocity *v*_*λ*_ = *λ*/*T*_*λ*_. For each pulse repetition *A*_*j*_ and *T*_*λ*_ can be varied. Pulse reversal (${\lambda }_{{{{\rm{x}}}}}^{-1}$ and ${\lambda }_{{{{\rm{y}}}}}^{-1}$) achieves reverse electron transfer.

From these pulses *λ*_L→R_ and ${\lambda }_{{{{\rm{R\to L}}}}}={\lambda }_{{{{\rm{L\to R}}}}}^{-1}$ are derived to transition between the idle and readout state (Fig. 1c).

The T-junction specific atomic pulse ${\lambda }_{{{{\rm{JR}}}}}^{1}$ transfers the charge occupancy from QD X_17_ into QD Y_0_ (Fig. 1d, finite-element simulation in Fig. 1f with details in Supplementary Note I). The transfer is realized by coordinated conveyor-mode shuttling with a drive amplitude *A*_J_ and velocity *v*_J_ in the x- and y-shuttle. This includes halting and reversing both shuttle lanes appropriately at *T*_*λ*_/4 intervals (Fig. 1d), but otherwise signal bandwidth requirements are similar to ${\lambda }_{{{{\rm{x}}}}}^{1}$ and ${\lambda }_{{{{\rm{y}}}}}^{1}$. We define the analogue atomic pulse transferring the charge occupancy directly from QD X_18_ into QD Y_0_, ${\lambda }_{{{{\rm{JL}}}}}^{1}$, differing only by the distance and direction of conveyor-mode shuttling in the x-shuttle (Supplementary Fig. 2). Of particular note is that the x- and y-shuttle need not be operated simultaneously during junction transfer.

All finally composed pulses are either led or followed by a ROI pulse (Fig. 1g), to recalibrate the SET current levels during the readout process (see methods). To clear the otherwise fully depleted shuttle lanes from remaining charges of previous experiments, we apply a flush pulse similar to ref. ^23^ (Supplementary Fig. 6a) at irregular intervals using *A*_x_ = *A*_y_ = *A*_J_ = 260 mV.

### Single electron shuttling

The main focus of this work is shuttling and routing across a T-junction, which first requires the calibration of other aspects of device operation. First, to access the junction between the x- and y-shuttle, we reproduce the linear shuttling as shown in ref. ^23^ in the x-shuttle. Because no charge sensor is implemented directly at the densely gated center of the junction, we investigate the initial success of routing across the T-junction by continuing to shuttle in the y-shuttle after transfer, until the shuttled charge encounters the upper end of the y-shuttle, which can be detected.

**Fig. 2:**
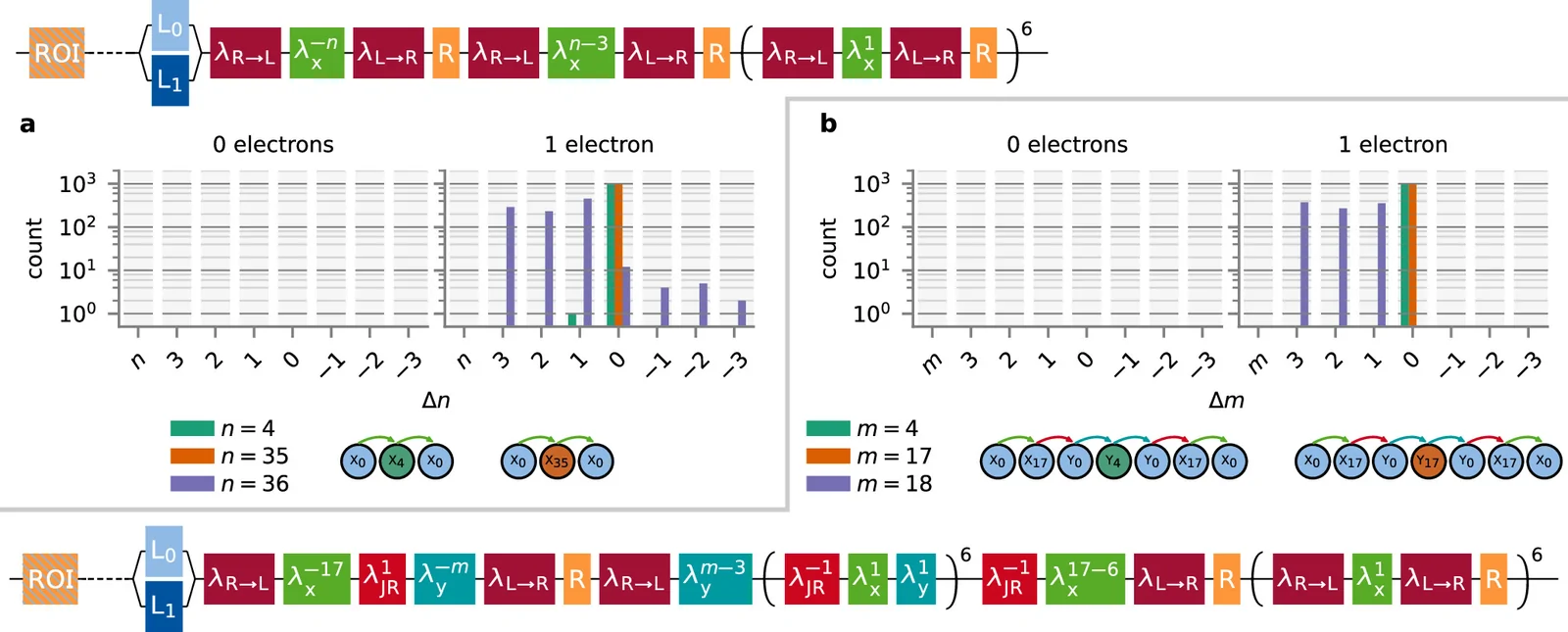
Verifying shuttling in the x- and y-shuttles. Applied pulses and readout histograms with one and zero electrons loaded, the latter as reference pulse. Branching notation in the pulses indicate that the whole pulse is repeated once for each branch, with the exception of the ROI-pulse separated by dashed lines, which is only performed for the first branch repetition. Histograms show the sum of QD X_0_ occupancy readout counts separated for the pulse branches with either one electron or zero electrons loaded. The QDs along the shuttle paths are shown next to the legends. *n*_total_ = 1000, *A*_x_ = *A*_y_ = *A*_J_ = 260 mV and *v*_*λ*_ = *v*_J_ = 270 mm s^−1^ for all measurements. Results for *v*_*λ*_ = *v*_J_ = 28 mm s^−1^ shown in Supplementary Fig. 3. **a** Shuttling in the x-shuttle. **b** Shuttling in the x-shuttle up to QD X_17_ followed by y-shuttle.

First we verify shuttling in the x-shuttle lane and thus shuttle straight through the T-junction employing the pulse shown in Fig. 2a. The pulse shuttles the initialized charge from QD X_0_, close to the right SET, into QD X_*n*_ and back to the right SET. During the pulse we perform readout of QD X_0_ to verify that the charge was shuttled away and finally returns to QD X_0_, shuttling for three periods more than required to return the charge. To check whether the electron can reach the end of the x-shuttle, we probe the effect of shuttling beyond the end of the x-shuttle similar to ref. ^23^. We perform seven readouts, three shuttling periods each before and after we expect the charge to return to detect unexpectedly advanced or delayed charge and one readout when the charge is expected. These readouts are labeled in the resulting data by their *Δn* value, which is Δ*n* = *n* for the first readout, when we expect the charge to be farthest from the SET and Δ*n* ∈ [ −3, 3], indicating the readouts relative to the expected Δ*n* = 0 readout (Δ*n* > 0 and Δ*n* < 0 corresponding to advanced or delayed charge, respectively). We apply this pulse with zero electrons loaded as a reference and then, immediately afterwards, with one electron to ensure the measured electron occupancy is only a consequence of the deliberate initialization of QD X_0_. We set the drive amplitude to *A*_x_ = *A*_y_ = 260 mV and investigate two nominal instantaneous shuttle velocities *v*_*λ*_ = 28 mm s^−1^ and *v*_*λ*_ = 270 mm s^−1^.

With few exceptions, no electrons are detected if zero electrons are loaded, as expected. When initializing QD X_0_ with one electron, that electron is observed as expected in readout Δ*n* = 0 when attempting to shuttle for *n* ∈ {4, 35} during almost all repetitions (Fig. 2a). For *n* = 36, far fewer electrons are detected in readout Δ*n* = 0, while many electrons are detected earlier in readouts Δ*n* ∈ {1, 2, 3}. This is our fingerprint that the electron actually reaches the left end of the x-shuttle (QD X_35_) because this deviation is a consequence of the finite size of the x-shuttle, as for *n* = 36 the pulse attempts to shuttle just beyond the last QD formed. Due to the low voltage applied to TLB1, electrons present in the moving potential encounter a large barrier at the end of the device, where the mobile QD confining the electrons continuously decreases in size until it ceases to be defined. The following redistribution of charge into the neighboring available quantum dots causes the electrons to be mostly advanced by one or more periods, as can be seen in the data, implying linear shuttling up to QD X_35_ has been successful (Fig. 2a). In few cases, but only for *n* = 36, electrons are detected later than expected (Δ*n* ∈ { − 1, − 2, − 3}). While electrons would be delayed if ${\lambda }_{x}^{1}$ pulses fail, the detection of these events only when the electrons are expected to reach the end of the x-shuttle implies a different mechanism. We attribute this observation to electrons shuttled or tunneled into the disordered potential barrier at the end of the shuttle being freed by repeated application of ${\lambda }_{x}^{1}$ pulses.

We apply the same technique to confirm successful inter-lane junction transfer (*v*_J_ = *v*_*λ*_, *A*_J_ = *A*_x_ = *A*_y_) by continuing to shuttle in the y-shuttle afterwards, until the outer end of the y-shuttle is reached (Fig. 2b). We substitute the Δ*n* notation to identify the nominal position of the electron with its analogue Δ*m* for this shuttle path. First, we shuttle the initialized charge from QD X_0_ to QD X_17_, the last QD before the junction from the right, perform the junction transfer, shuttle the charge from QD Y_0_ to QD Y_*m*_, perform a readout (Δ*m* = *m*), return to QD X_0_ while ensuring the transfer of the three QDs leading and following the initialized QD through the junction and reading-out their charge occupancy (Δ*m* ∈ [ −3, 3]). Of particular note is the pulse asymmetry between the transfer to and back from QD Y_*m*_, ensuring that a failure of inter-lane junction transfer during ${\lambda }_{{{{\rm{JR}}}}}^{1}$ is detected, as the electron loaded would appear early for readout.

For this x-y-shuttle sequence, we observe a similar result as for the pure x-shuttle sequence (Fig. 2a). The pulse for *m* ∈ {4, 17} returns the electron reliably (Δ*m* = 0), but *m* = 18 does not, indicating that we reached the top end of the y-shuttle (Fig. 2b). This observation matches the expectation given by the device geometry and we conclude that inter-lane junction transfer shuttling is achieved. We evaluate the pulse fidelity *F*_total_ as defined in the methods section (results noted in Table 1), which does not significantly depend on the distance shuttled.

**Table 1:** Shuttle fidelities at *A*_x_ = *A*_y_ = *A*_J_ = 260 mV and *v*_*λ*_ = *v*_J_ = 270 mm s^−1^

shuttle path	*F*_total_ (%)	*F*_1_ (%)
X_0_X_4_X_0_	$99.{9}_{-0.5}^{+0.1}$	
X_0_X_35_X_0_	$100.{0}_{-0.4}^{+0.0}$	
X_0_X_17_Y_0_Y_4_Y_0_X_17_X_0_	$100.{0}_{-0.4}^{+0.0}$	
X_0_X_17_Y_0_Y_17_Y_0_X_17_X_0_	$100.{0}_{-0.4}^{+0.0}$	
${{{{\rm{X}}}}}_{0}{\left({{{{\rm{X}}}}}_{16}{{{{\rm{Y}}}}}_{1}{{{{\rm{X}}}}}_{19}\right)}^{1{0}^{5}}{{{{\rm{X}}}}}_{0}$	$100.{0}_{-0.4}^{+0.0}$	${100.000000}_{-4\times 1{0}^{-6}}^{+0}$
${{{{\rm{X}}}}}_{0}{\left({{{{\rm{X}}}}}_{16}{{{{\rm{Y}}}}}_{1}{{{{\rm{X}}}}}_{19}\right)}^{1{0}^{6}}{{{{\rm{X}}}}}_{0}$	$99.{8}_{-0.9}^{+0.2}$	${99.9999998}_{-9\times 1{0}^{-7}}^{+2\times 1{0}^{-7}}$

Shuttle paths are indicated as sequences of key QDs traversed in time-order. ${{{{\rm{X}}}}}_{0}{\left({{{{\rm{X}}}}}_{16}{{{{\rm{Y}}}}}_{1}{{{{\rm{X}}}}}_{19}\right)}^{1{0}^{6}}{{{{\rm{X}}}}}_{0}$ with *A*_J_ = 100 mV yields ${F}_{1}={100.0000000}_{-9\times 1{0}^{-7}}^{+0}\,\%$.

Using this method a significant deviation of *F*_total_ from 100 % could not be determined in a reasonable amount of measurement time, due to the duration of atomic pulses L_0_, L_1_ and R_CC_.

**Fig. 3:**
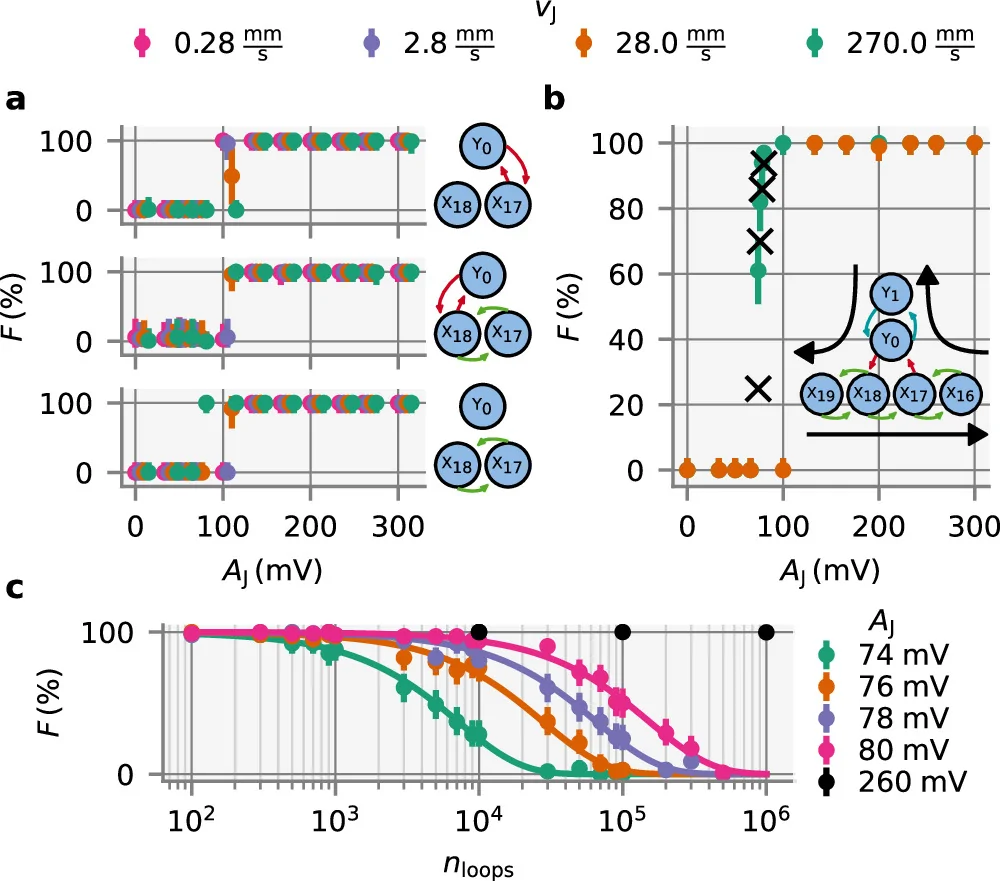
Parameter variation and charge looping within the T-junction. *n*_total_ = 100, *A*_x_ = *A*_y_ = 260 mV and *v*_*λ*_ = 270 mm s^−1^ for all measurements if not otherwise indicated. **a** Pulse fidelity at different drive amplitudes *A*_J_ and shuttle velocities *v*_J_ during shuttling in the junction. Additional shuttling into the y-shuttle is not shown schematically. Data points offset in *A*_J_ by 5 mV per *v*_J_ value starting from *v*_J_ = 0.28 mm s^−1^. Error bars are $4{\sigma }_{{F}_{{{{\rm{total}}}}}}^{\pm }$. **b** Fidelity at different drive amplitudes *A*_J_ and shuttle velocities *v*_J_ during looped inter-lane junction transfer for *n*_loops_ = 10,000. Black crosses are interpolated from *F*_1_ extracted in (**c**). Error bars are $1{\sigma }_{{F}_{{{{\rm{total}}}}}}^{\pm }$. **c** Pulse fidelity for different numbers of loops *n*_loops_ at *v*_J_ = 270 mm s^−1^ and select drive amplitudes *A*_J_ for the same pulse as (**b**). *n*_total_ = 1000 and *n*_total_ = 490 for *A*_J_ = 260 mV at *n*_loops_ = 10^5^ and *n*_loops_ = 10^6^, respectively. Error bars are $1{\sigma }_{{F}_{{{{\rm{total}}}}}}^{\pm }$.

When shuttling in the junction, three main shuttle paths exist, each with its own range of possible drive amplitudes and shuttle velocities. To explore the available parameter space, we apply several pulses that first shuttle the initialized QD close to the junction using known good drive amplitudes *A*_x_ = *A*_y_ = 260 mV and shuttle velocity *v*_*λ*_ = 270 mm s^−1^ and then either route the initialized QD into the y-shuttle lane using ${\lambda }_{{{{\rm{JR}}}}}^{1}$ or ${\lambda }_{{{{\rm{JL}}}}}^{1}$ or shuttle from QD X_16_ to QD X_18_ using ${\lambda }_{{{{\rm{x}}}}}^{-2}$, while varying the drive amplitude *A*_J_ at shuttle velocity *v*_J_ (Fig. 3a). Note that we set *A*_x_ = *A*_J_ and *v*_*λ*_ = *v*_J_ for the final path when shuttling from QD X_16_ to QD X_18_ only. The full pulses used here are shown in Supplementary Fig. 4. The pulse fidelities observed remain close to 100 % for *A*_J _≥ 133 mV for ${\lambda }_{{{{\rm{JR}}}}}^{1}$, ${\lambda }_{{{{\rm{JL}}}}}^{1}$ and ${\lambda }_{{{{\rm{x}}}}}^{-2}$, with only a minor dependence of the minimum reliable drive amplitude on the instantaneous shuttle velocity during junction transfer shuttling *v*_J_. While the cable bandwidth limits the investigated *v*_J_ to one order of magnitude lower than suggested for spin-coherent shuttling^28^, we observe no significant reduction of pulse fidelity up to the maximum possible *v*_J_. The drive amplitudes allowing for successful inter-lane junction transfer shuttling are similar to the required drive amplitude for linear shuttling in ref. ^23^, performed on a different device design, albeit on a nominally identical heterostructure.

### Charge looping within the T-junction

As the previous pulses transfer the initialized charge through the junction only two times per pulse, we expect their fidelity to be dominated by charge state preparation and readout (SPAM) errors and possibly by errors occurring when shuttling along the x- and y-shuttle lanes. Additionally, charge initialization and readout contribute the majority of measurement time, thereby limiting the total number of repetitions. Hence, these pulses are not able to resolve the small infidelity of inter-lane junction transfer (${\lambda }_{{{{\rm{JR}}}}}^{\pm 1}$ and ${\lambda }_{{{{\rm{JL}}}}}^{\pm 1}$). Therefore, we modify the pulse such that the initialized charge is instead shuttled into QD X_16_ using again the known good drive amplitudes *A*_x_ = *A*_y_ = 260 mV. Then, we loop the charge *n*_loops_-times within the T-junction, following the looped shuttle path indicated in Fig. 3b (inset) before returning to QD X_0_, performing a readout procedure identical to the previous pulses (results noted in Fig. 3b, full pulse shown in Supplementary Fig. 4d).

During each loop, the nominally empty shuttle lane, which the electron is expected to have left due to the applied junction transfer atomic pulse (${\lambda }_{{{{\rm{JR}}}}}^{1}$ or ${\lambda }_{{{{\rm{JL}}}}}^{-1}$) is operated for 25 periods (${\lambda }_{{{{\rm{x}}}}}^{25}$ or ${\lambda }_{{{{\rm{y}}}}}^{-25}$). This large number of additional shuttle pulses provides us high confidence that the electron is shuttled away from the center of the T-junction if the junction transfer atomic pulse fails. Thus, the detection of a failed ${\lambda }_{{{{\rm{JR}}}}}^{1}$ or ${\lambda }_{{{{\rm{JL}}}}}^{-1}$ atomic pulse is ensured. As *n*_loops_ increases, the ratio of electron loading events and readouts (i.e. SPAM) against the number of shuttle atomic pulses decreases, rendering the resulting fidelity more sensitive to shuttle errors occurring during looping within the T-junction. This strategy allows us to measure the shuttle fidelity of looping within the T-junction with extremely high accuracy.

The fidelity per charge loop *F*_1_ (see methods) does not significantly drop below 100 % for junction transfer drive amplitudes *A*_J _≥ 133 mV. The charge loops are repeated for select *A*_J_ at *v*_J_ = 270 mm s^−1^ as a function of *n*_loops_ and we fit ${F}_{{{{\rm{total}}}}}({n}_{{{{\rm{loops}}}}})={F}_{1}^{{n}_{{{{\rm{loops}}}}}}$ to the resulting data (Fig. 3c, fits indicated as solid lines). The data follow these fits well, indicating statistical independence of failure of junction transfer per loop. As a sanity check, we calculate *F*_total_(*A*_J_, *n*_loops_ = 10,000) from the determined *F*_1_(*A*_J_) (crosses in Fig. 3b), which agree with the directly measured results (dots in Fig. 3b) at *v*_J_ = 270 mm s^−1^. The remaining deviation between the direct measurement and this check remains below the usual voltage drift in the device for these comparatively long measurements.

To further resolve the transfer fidelity, we repeat the experiment at *A*_J_ = 260 mV for large *n*_loops_ with complete results noted in Table 1. We additionally reduce the junction transfer drive amplitude to its approximate reliable minimum *A*_J_ = 100 mV, which at *n*_loops_ = 10^6^ and *v*_J_ = 270 mm s^−1^ yields ${F}_{1}={100.0000000}_{-9\times 1{0}^{-7}}^{+0}\,\%$, not significantly differing from the result at *A*_J_ = 260 mV.

We can feed the applied pulses back to the simulation to investigate the expected quantum states during shuttling with *A*_J_ = 260 mV and *A*_J_ = 100 mV (Supplementary Note I). The simulation indicates that the electron is continuously moved around the junction.

### Multiple electron operation

Using parameters *A*_x_ = *A*_y_ = *A*_J_ = 260 mV and *v*_*λ*_ = *v*_J_ = 270 mm s^−1^ for which a single electron is reliably shuttled across the whole T-junction device, we demonstrate that multiple electrons can be distributed across the device and shuttled using the same simple set of atomic pulses (analogous to the linear shuttle device in ref. ^23^). Independent control of the x- and y-shuttle lane additionally allows us to change the order of electrons within the x-shuttle.

**Fig. 4:**
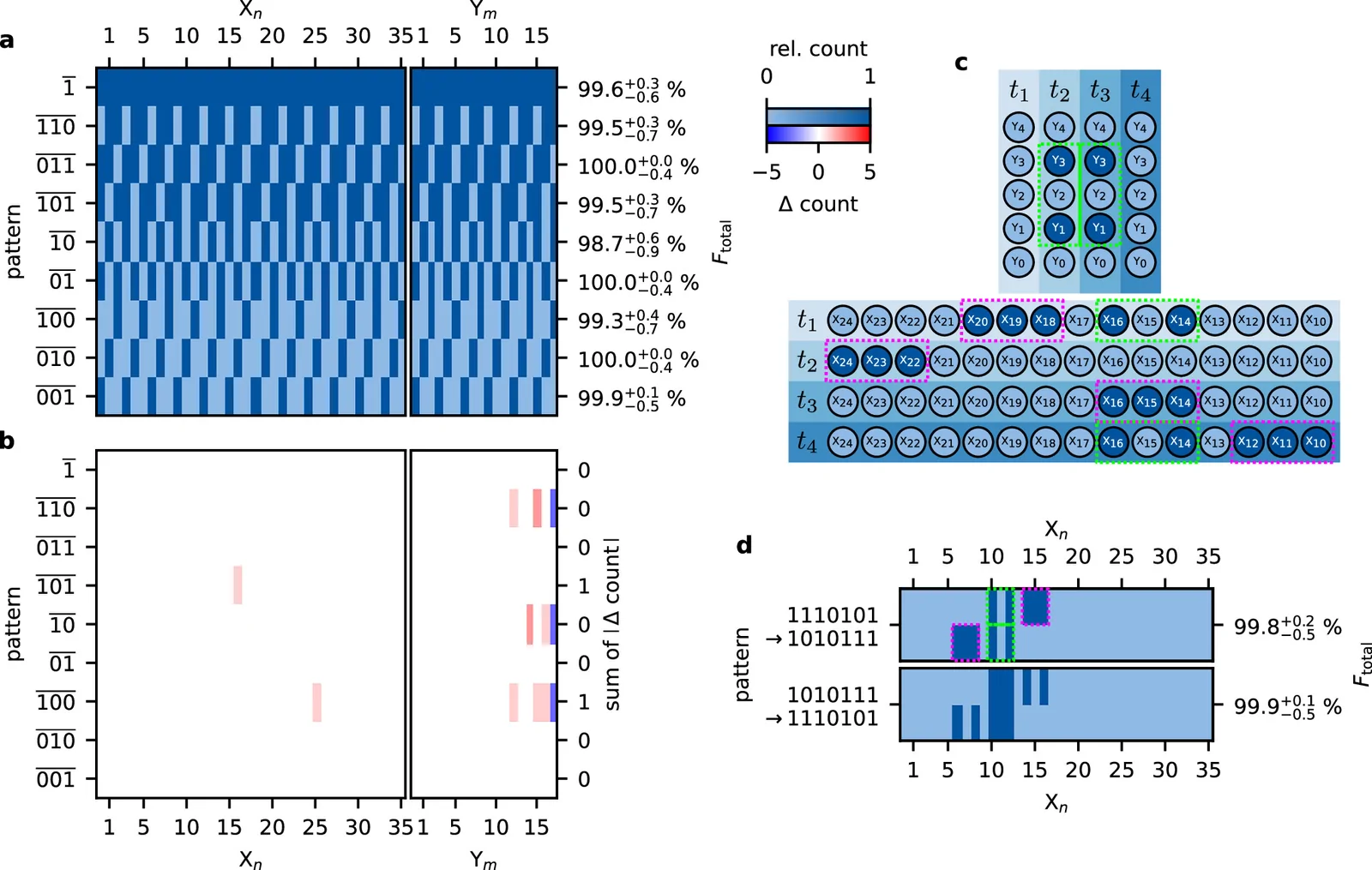
Multiple electron operation. Color bars apply to (**a**), (**b**) and (**d**). Blue color bar: average QD occupancy over pulse repetitions. Blue-red color bar: Sum of QD occupancy deviating from expectation over pulse repetitions. *n*_total_ = 1000 for each electron pattern and SWAP. If two electrons are detected in the same QD, the total result of the pulse is excluded from occupancy counts while remaining included in the calculation of *F*_total_. **a** Results for loading periodic electron occupancy patterns into the x- and y-shuttle. The patterns are held in both channels simultaneously. Overline notation to the left of the plot identifies the periodic patterns. The total fidelity of each pattern is displayed to the right. **b** Deviation from expectation in (**a**). Sum of absolute sum of deviations over all electron counts in each repetition over all repetitions, is noted to the right. **c** Expected charge configuration of a section of the x- and y-shuttle when performing an electron pattern SWAP. Dark blue QD: one electron in QD. Light blue QD: no electron in QD. The leading and following patterns are outlined in magenta and green, respectively. Labels *t*_1_ through *t*_4_ indicate four time steps during the pulse. *t*_1_: The two sequential patterns are shuttled to the junction. *t*_2_: The following pattern is transferred into the y-shuttle. *t*_3_: The leading pattern is retracted. *t*_4_: The following pattern is retrieved and the pattern SWAP is complete. **d** Results for pattern SWAP. Patterns are highlighted matching (**c**). Pulse fidelities are shown to the right. The deviation from expectation is noted in Supplementary Fig. 5.

First, we show that we can initialize an electron pattern - the filling (empty or singly occupied) of multiple QDs in the device. We start by loading an electron pattern into the right half of the x-shuttle, and then shuttle the pattern via repeated ${\lambda }_{{{{\rm{JR}}}}}^{1}{\lambda }_{{{{\rm{x}}}}}^{-1}{\lambda }_{{{{\rm{y}}}}}^{-1}$-pulses into the y-shuttle, load a new pattern into the entire x-shuttle and finally read-out both shuttle lanes in reverse order (Fig. 4a). An example for a full pulse is shown in Supplementary Fig. 6c. In order to verify whether arbitrary patterns can be prepared, we load different periodic patterns varying between full electron filling and 1:2 ratios of electron-filled and empty QDs. These periodic patterns are chosen with electrons having unoccupied and occupied neighboring QDs such that we can investigate whether the filling of a neighboring QD leads to specific errors, e.g. one electron tunneling to an unoccupied neighboring QD. Therefore, the deviation of the number of electrons counted from the expected count in a QD as well as the absolute sum of these deviations in a pulse repetition can be used to detect such events. The sums over all repetitions of these two values are indicated in Fig. 4b. Note that in particular for patterns with high QD occupancy, e.g. $\overline{1}$, it is impossible to differentiate unintentional electron tunneling from a SPAM error during L_1_ resulting in the detection of two electrons in the same QD. Therefore, we eliminate all pulses with two electrons detected in any QD from the QD occupancy counts. In the calculation of *F*_total_, we count a pulse as successful (failed) if one or two electrons are detected in a QD with one electron (zero electrons) expected (see methods).

Tunneling across neighboring QDs is only observed in few repetitions and only from QD Y_17_ into nearby QDs, likely due to the large potential barrier present at the edge of the y-shuttle. The remaining errors observed are the occasional detection of additional electrons during readouts where no electrons were expected. We attribute this error to a likely too transparent barrier between QD X_0_ and the adjacent electron reservoir during initialization, combined with an uncompensated drift in the calibrated L_0_ atomic pulse, causing unintentional loading of electrons (i.e., SPAM errors). It is especially remarkable that even shuttling and routing of 54 electrons in the two independent shuttle lanes simultaneously yields pulse fidelities of *F*_total_ ≈ 99 %.

An interesting feature of the T-junction is the ability to perform a native spin-SWAP gate by physically swapping electron positions. The fidelity of the electron exchange-based spin-SWAP gate often suffers from the diabatic switching of the exchange interaction^40^ and is one of the two-qubit gates with typically low fidelity. Since we have not implemented a spin detector in the T-junction yet, we emulate the SWAP operation by swapping the electron order of two consecutively prepared electron patterns utilizing the independent control of the x- and y-shuttle (Fig. 4c with the pulse shown in Supplementary Fig. 6d). We observe pulse fidelities *F*_total _≥ 99.8 % (Fig. 4d) and no significant difference in pulse fidelity dependent on the initial order of the two prepared patterns is apparent. This result serves as an indication that a future spin-SWAP gate by routing within a T-junction could become an attractive alternative to the exchange-based SWAP and can be naturally implemented in two-dimensional shuttling architectures such as proposed in ref. ^15,29^.

## Discussion

We demonstrate single electron routing across a conveyor-mode shuttling based T-junction in Si/SiGe. In general, the charge shuttle fidelity is high and the shuttle velocity of 270 mm s^−1^ is 2000 times higher than achieved in the longest linear shuttle device demonstrated so far^23^. Already at this velocity, a spin phase error of  < 10 % for coherent electron spin shuttling across the entire device could be achieved at a spin ensemble coherence time of ${T}_{2}^{*}=20\,\mu {{{\rm{s}}}}$ and accounting for motional narrowing^28^. In particular, a high charge transfer fidelity of $F={100.0000000}_{-9\times 1{0}^{-7}}^{+0}\,\%$ in the center of the T-junction is determined. This is remarkable, as most gates of the T-junction are connected and no tuning of voltages applied to individual gates is performed. Such fine-tuning is common for state-of-the-art qubit devices, posing a challenge for device scalability. We operate the T-junction device that consists of 54 QDs by only eleven voltage channels, three of which required solely for charge initialization and readout by an SET. In total, we need only 5 DC voltages, including one voltage to form the barriers for the top and left end of the shuttle lanes and 4 voltages to form the SET. Eight short atomic voltage pulses, from which we easily compile complex pulses by an arbitrary waveform generator, are sufficient to operate the whole T-junction device. These pulses can control up to 54 electrons with arbitrary single electron fillings of all QDs with high accuracy. In particular, we use our T-junction to swap electron positions with fidelity exceeding the fidelity of an exchange-based SWAP gate commonly used for spin qubits.

Controlling positions of many single electrons with simple voltage pulses in two dimensions has important implications, especially addressing the scalability of spin-based quantum computation. The voltage tuning is simple and fast and fewer signal generators are needed. In fact, our T-junction has been operated in two thermal cycles without need of voltage fine-tuning. The number and simplicity of signal channels is crucial for the co-integration of cryo-electronics^41^, which in turn is a prerequisite for scalable architectures^42^. Our work might guide these circuit designs^43^.

This high level of single electron control might also be exploited for novel low-power classical electronics and dense memories operating with single electrons in QDs below 1 K. It might also become useful in metrology for current standards based on electron counting. It is also promising for fault-tolerant quantum computing with spin qubits^31^, for which millions of electrons have to be positioned in a two-dimensional matrix of shuttle lanes^29^. In short lanes, spin-qubit shuttling with high velocity^21^ and spin manipulation^44^ has been demonstrated. While it is significant to design and demonstrate devices for conveyor-mode charge routing with minimal control complexity, note that spin-qubit shuttling in Si/SiGe requires material progress mainly on homogeneous enhancement of valley-splitting across large areas of the devices^26^.

## Methods

### Device fabrication

The device is fabricated on an undoped Si_0.70_Ge_0.30_/Si/Si_0.70_Ge_0.30_ heterostructure, which is grown on multiple buffer layers on a (001) CVD-grown silicon wafer. All silicon in this experiment is of natural isotope composition. The tensile strained, nominally 10nm thick Si layer forms a quantum well hosting a two-dimensional electron gas. The upper Si_0.70_Ge_0.30_ layer has a nominal thickness of 30 nm and is capped with 2 nm of Si. The lower Si_0.70_Ge_0.30_ layer has a nominal thickness of 2 μm with an intermediate, chemically-mechanically polished step after 1μm. Beneath the buffer, a nominally 3 μm thick linearly graded Si_1−*x*_Ge_*x*_ (*x* ∈ [0.05, 0.3]) buffer is deposited on the substrate silicon wafer.

Ohmic contacts are fabricated by phosphorus ion implantation and thermal activation at 730 ^∘^C for 30 s. The three metal gate layers are made of a Ti/Pt stack (5 nm and 15 nm/22 nm/29 nm, respectively), shaped by 100 keV electron-beam lithography (schematics of gate layers in Supplementary Fig. 7) and lift-off, isolated from each other and the substrate by amorphous, 10 nm thick layers of Al_2_O_3_, fabricated by atomic layer deposition.

### Device design and operation

The lowest gate layer is comprised of three large, grounded screening gates (ScrTL, ScrTR, ScrBR) transversely confining two  ≈ 5 μm and  ≈ 10 μm long one-dimensional electron channels (1DECs) joining at a 90^∘^ angle in the device center, while confinement into quantum dots along the channels is achieved via 4 claviature gates per channel in gate layers two (LS2, RS2, TS2, LS4, RS4 and TS4) and three (LS1, RS1, TS1, LS3, RS3 and TS3). Six individual gates (JLS, JLP, JP, JRP, JRS and JTS) could enable independent control in the critical junction center. The electron channels are terminated towards the edges of the device with three individual gates each (TLB1/2, TLP, TRB1/2, TRP, TTB1/2 and TTP), beyond which a single electron transistor (SET) each is formed. The right SET serves as an electron reservoir and proximal charge detector^45^. In total 218 claviature gates span the 1DECs, forming the two shuttle lanes.

Due to the periodic gating defining the shuttle lanes, several gates in each lane are grouped into gate sets, following the color coding in Fig. 1. To all gates within one gate set the same voltage is applied. The eight periodic gate sets are: S_x,1_ = TLP, LS3, JRP and RS1; S_x,2_ = TLB2, LS4, JLS, JRS, TRB2 and RS4; S_x,3_ = LS1, JLP and RS3; S_x,4_ = LS2, JP and RS2; S_y,1_ = TTP and TS3; S_y,2_ = TTB2 and TS4; S_y,3_ = TS1; S_y,4_ = TS2 and JTS. Gates belonging to these gate sets, as well as TRB1, TRP and RB1 are controlled by voltage pulses.

### Experimental setup

The device is mounted on the mixing chamber stage of an Oxford Instruments wet dilution refrigerator at a temperature of  ≈ 30 mK. Signals inside the refrigerator are carried to the sample via custom-made high density flexible flat cables with a bandwidth on the order of 1 MHz. This bandwidth allows for experimentation close to the frequency range used for spin-coherent single electron shuttling^24^. At room temperature, voltages applied to gates that are held constant are generally applied using custom-made low noise digital to analog converters. Voltages are applied to pulsed gates using Zürich Instruments HDAWG arbitrary waveform generators. The two ohmic contacts of the SET used for readout are connected to room-temperature bias-tees allowing for an offset voltage as well as a modulated voltage to be applied and a modulated current to be measured. For the results presented here, the offset voltage is set to 0V while the modulated voltage on the order of 100 μV is applied using the output of a Zürich Instruments MFLI lock-in amplifier. The modulated current is then converted to a voltage by a Basel Precision Instruments transimpedance amplifier, which is measured using the lock-in amplifier. For ease of integration, the demodulated signal is converted to an appropriately scaled voltage output by the lock-in amplifier, buffered by a Stanford Research Systems SR560 preamplifier and acquired using an AlazarTech ATS9440 waveform digitizer.

### Fidelity calculation and error estimation

We consider the result of a shuttle pulse containing several atomic readout pulses a success if electrons are detected during and only during the readout pulses for which we expect electrons to be detected. Thus, a pulse in which an electron is detected where no electron is expected is considered a failed pulse. If *n*_s_ is the number of successful pulse repetitions and *n*_total_ is the total number of repetitions, the pulse fidelity *F*_total_ is defined as:

2 $${F}_{{{{\rm{total}}}}}=\dfrac{{n}_{{{{\rm{s}}}}}}{{n}_{{{{\rm{total}}}}}}.$$

The generally asymmetric uncertainty on *F*_total_ is determined from the two-sided, 95% Clopper-Pearson confidence interval $\left[{F}_{{{{\rm{total}}}},\,95\,\%}^{-},{F}_{{{{\rm{total}}}},\,95\,\%}^{+}\right]$ as ${\sigma }_{{F}_{{{{\rm{total}}}}}}^{\pm }={F}_{{{{\rm{total}}}},\,95\,\%}^{\pm }-{F}_{{{{\rm{total}}}}}$. The value of *F*_total_ and its uncertainty is denoted as ${({F}_{{{{\rm{total}}}}})}_{{\sigma }_{{F}_{{{{\rm{total}}}}}}^{-}}^{{\sigma }_{{F}_{{{{\rm{total}}}}}}^{+}}$.

Occasionally, we observe from the SET current level that two electrons instead of one expected electron are detected. For these rare cases, we still label the repetition as successful, as we attribute the observation to the unintended loading of an additional electron presumably during the intended electron loading, which constitutes a SPAM error. The detection of an additional electron in the QD, in which one electron is expected due to the application of L_1_, is explained by the conveyor-mode shuttle operating with doubly occupied QDs. While the determination of this observation as a success reduces the impact of SPAM errors on the pulse fidelity, it is stricter on the fidelity of charge shuttling, as the confinement of two electrons in a QD is expected to be weaker and therefore more prone to shuttle errors than shuttling only one electron, e.g. one electron tunneling into a neighboring, unoccupied QD.

### Fidelity calculation for looping in the T-junction

The fidelity per charge loop *F*_1_ and the corresponding uncertainties ${\sigma }_{{F}_{1}}^{\pm }$ are calculated following:

3 $${F}_{1}={F}_{{{{\rm{total}}}}}^{\dfrac{1}{{n}_{{{{\rm{loops}}}}}}}$$

4 $${\sigma }_{{F}_{1}}^{\pm }={({F}_{{{{\rm{total}}}},\,95\,\%}^{\pm })}^{\dfrac{1}{{n}_{{{{\rm{loops}}}}}}}-{F}_{1}$$

These values are denoted as ${({F}_{1})}_{{\sigma }_{{F}_{1}}^{-}}^{{\sigma }_{{F}_{1}}^{+}}$.

### Readout procedure

During each readout (i.e. application of R_CC_), the current through the SET is monitored and averaged in the time interval *t*_RO_. Due to the capacitive coupling of the charge present in QD X_0_ in the readout state, the chemical potential of the SET’s sensing is altered, leading to proximal charge detection by the current across the SET. We can use the resulting current to readout the number of electrons occupying QD X_0_ using the following procedure: First, the current background as well as current thresholds allowing determination of the number of electrons in QD X_0_ are extracted. The background and thresholds are calculated block-wise over differing numbers of repetitions to accommodate changing system noise and SET current drift. The background in one pulse repetition is calculated by averaging the measured current during all readouts in each pulse repetition and then averaged over the current block of repetitions. The background is subtracted from the measured current. The reference readouts directly following the L_1_ and L_0_ atomic pulses within the ROI pulse contained in each pulse repetition are used to determine the current thresholds discriminating the number of electrons in QD X_0_. To make the observed threshold robust against unintentionally incorrect charge occupancy of QD X_0_, we remove the 5 % largest and smallest readout currents each from the calculation. The threshold discriminating between zero and one electron is set as the mid-point between the average current in the reference readouts and then averaged over the current block of repetitions.

It becomes apparent that in  ≈ 0.04 % of readouts, two electrons are unexpectedly detected in QD X_0_. While a clear distinction between outliers in the current during the detection of one electron and the detection of two electrons can not always be made, we still attempt to make this discrimination. The corresponding threshold discriminating between one and two electrons detected is set $4\cdot {\sigma }_{{{{{\rm{L}}}}}_{1}}$ beyond the average current during the reference readout after L_1_, where ${\sigma }_{{{{{\rm{L}}}}}_{1}}$ is the population standard deviation of the average measured currents during this readout, taken over the current block of repetitions.

## Supplementary information

Supplementary Information

Peer Review file

Description of Additional Supplementary Files

Supplementary Movie 1

Supplementary Movie 2

## Data Availability

The data generated in this study have been deposited in the Zenodo database under accession code 18032292.
