# Supplementary material for: Conveyor-mode electron shuttling through a T-junction in Si/SiGe
Source: Nat Commun. 2026 Nov 5;17:11453. doi: 10.1038/s41467-026-78805-1 (PMC13740304; doi:10.1038/s41467-026-78805-1)
Supplement: Supplementary file 1 [file 41467_2026_78805_MOESM1_ESM.pdf]

Supplementary Information - Conveyor-mode electron shuttling through a T-junction in Si/SiGe

Max Beer [1,2] Ran Xue,[1] Lennart Deda,[1] Stefan Trellenkamp,[3] Jhih-Sian Tu,[3]
Paul Surrey,[1] Inga Seidler,[1] Hendrik Bluhm,[1,2] and Lars R. Schreiber [1,2,*]

[1] *JARA-FIT Institute for Quantum Information, Forschungszentrum Jülich GmbH and RWTH Aachen University, 52074 Aachen, Germany*
[2] *ARQUE Systems GmbH, 52074 Aachen, Germany*
[3] *Helmholtz Nano Facility (HNF), Forschungszentrum Jülich, Jülich, Germany*

SUPPLEMENTARY NOTE I: SIMULATION OF SHUTTLING IN THE T-JUNCTION

We simulate the wavefunction of an electron confined to a quantum dot (QD) in the device by solving the one-electron time independent Schroedinger equation on a two-dimensional grid on the gate-controlled instantaneous potential landscape at many time steps within a suitable region around the expected electron position. The total potential within the Si/SiGe quantum well for each time step is calculated in COMSOL Multiphysics® using a model of the device. During the calculation we disregard charge screening within the quantum well as the relevant device region is expected to be almost fully depleted. To verify continuous electron motion, we evaluate the expectation value of the ground state position $\langle x_i \rangle = \langle g|x_i|g \rangle$, where $x_i \in \{\mathrm{x}, \mathrm{y}\}$ is the x- or y-coordinate, and $|\mathrm{g}\rangle$ is the electron ground state. Analogous to Ref. [1], we also calculate the resulting orbital splitting $\mathrm{E_{orb}} = \langle \mathrm{e}|H|\mathrm{e}\rangle - \langle \mathrm{g}|H|\mathrm{g}\rangle$ between $|\mathrm{g}\rangle$ and the first excited state $|\mathrm{e}\rangle$, where H is the system Hamiltonian.

As the central device aspect, we simulate shuttling from QD $\mathrm{X_{16}}$ to QD $\mathrm{Y_0}$ using $A_\mathrm{x} = A_\mathrm{y} = A_\mathrm{J} = A$, twice using $A = 260\,\mathrm{mV}$ and $A = 100\,\mathrm{mV}$, respectively as well as similar DC voltages as used in the experiment. The results are shown in Supplementary Fig. 1a-c and Supplementary Fig. 1e-g, with probability densities for key time steps rendered in Supplementary Fig. 1d and Supplementary Fig. 1h. Renderings of full simulations are shown in Supplementary Movie 1 ($A = 260\,\mathrm{mV}$) and Supplementary Movie 2 ($A = 100\,\mathrm{mV}$).

Coinciding with the respective minimum in $\mathrm{E_{orb}}$ for both drive amplitudes, a fast increase in $\langle y \rangle$ is obtained. This increase is more pronounced for $A = 100\,\mathrm{mV}$ compared to $A = 260\,\mathrm{mV}$, however both $\langle x \rangle$ and $\langle y \rangle$ are continuous for both drive amplitudes indicating that electron transfer occurs without long-range tunneling (Supplementary Fig. 1a, e).

The minima in $\mathrm{E_{orb}}$ occur near $2T_\lambda$, decreasing to $\mathrm{E_{orb,min}} \approx 910\,\mu\mathrm{eV}$ and $\mathrm{E_{orb,min}} \approx 185\,\mu\mathrm{eV}$ for $A = 260\,\mathrm{mV}$ and $A = 100\,\mathrm{mV}$, respectively. Further inspection reveals the cause for this decrease as the lowered confinement when merging the QD containing the electron and the first QD of the vertical conveyor. As $\mathrm{E_{orb}}$ decreases, excitation into the excited state may occur, even at low shuttle velocities. If the occupancy of these states is uncontrolled and orbital relaxation occurs slowly or excitations occur often, spin state decoherence occurs, as the electron g-factor and valley splitting are expected to differ significantly between ground and excited states [2, 3], warranting further investigation. We estimate that realistic potential disorder, caused by e.g. charged defects in the semiconductor-oxide interface above the Si capping layer, could decrease $\mathrm{E_{orb,min}}$ into a problematic regime for $A = 100\,\mathrm{mV}$ (c.f. Ref. [1]).

When sufficiently far away from the junction center, a consistent primary confinement axis with respect to the shuttle direction is observed, when the electron is centered below a gate. For $A = 260\,\mathrm{mV}$, this axis is aligned longitudinally with the shuttle direction (indicating stronger confinement by shuttle gates) and for $A = 100\,\mathrm{mV}$ it is aligned transversely (indicating stronger confinement by screening gates). We refer to the initial and final excited states during the simulation as $|\mathrm{e_x}\rangle$ and $|\mathrm{e_y}\rangle$, respectively, and plot the projection of the instantaneous first excited state onto these states in Supplementary Fig. 1b and Supplementary Fig. 1f.

For $A = 260\,\mathrm{mV}$, the first excited states remain well aligned with the primary confinement axes, except for short lived rotations when the electron is centered between two gates. Using this metric, the moment of transfer between the x- and y-shuttle can be identified when the first excited state rotates by 90°, coinciding with a maximum in $\mathrm{E_{orb}}$.

For $A = 100\,\mathrm{mV}$, this rotation still occurs during this transfer, however similar rotations occur at several time steps as the confinement strength varies with the height of the gate the electron is currently under. Tuning drive amplitude and DC voltage separately for each gate layer is expected to suppress these additional rotations. Additionally, no

* lars.schreiber@physik.rwth-aachen.de

maximum in E_{orb} is observed in the moment of transfer due to the weak confinement in the junction center, as indicated by the low $E_{orb,min}$ and the non-circular electron probability density.

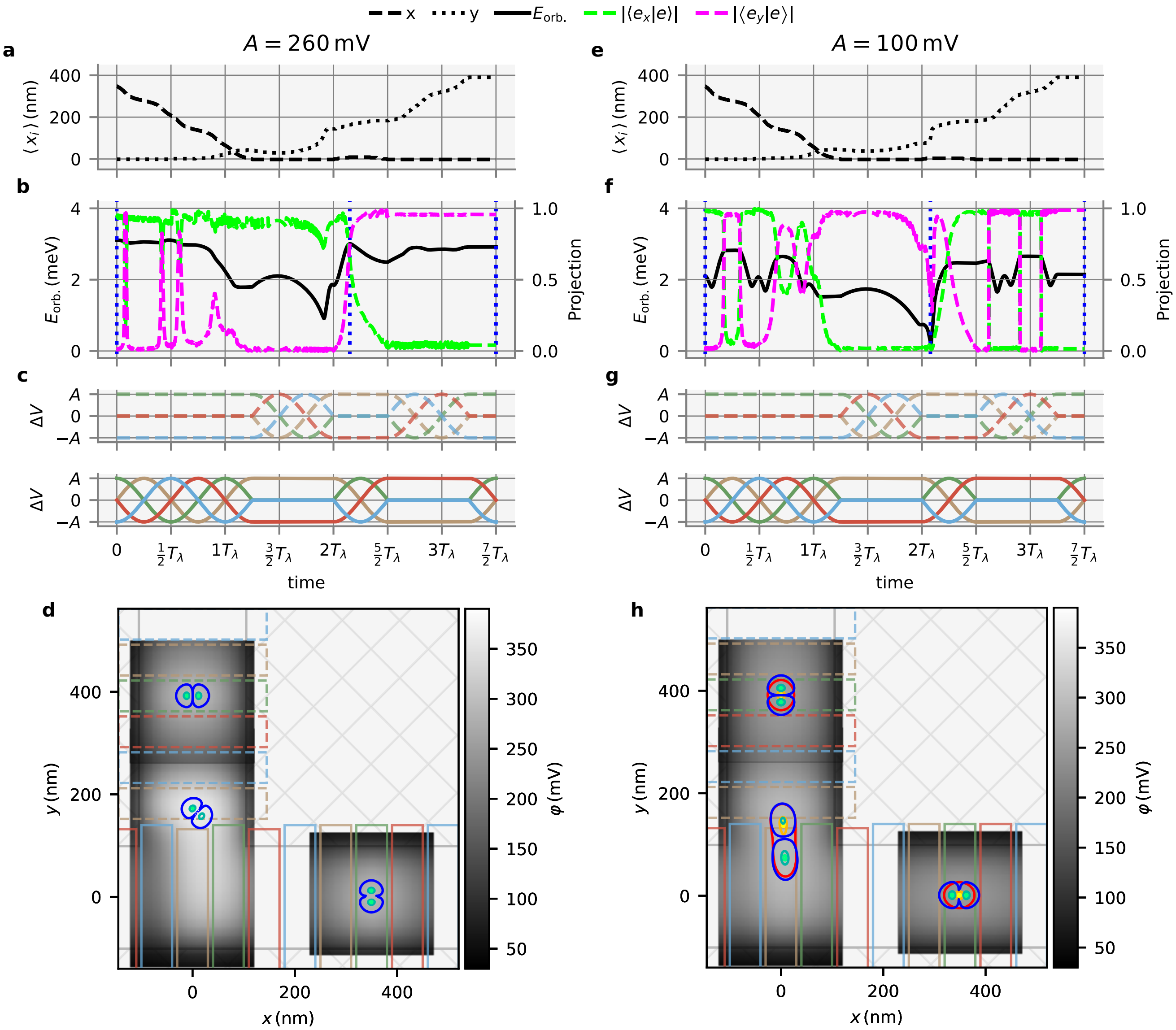

Supplementary Figure 1. **Additional simulation results for $A = 260\,\mathrm{mV}$ and $A = 100\,\mathrm{mV}$.** $A_x = A_y = A_J = A$. **a, e** Expectation value of electron x and y position in the ground state. **b, f** Orbital splitting and wavefunction projection during transfer. Blue markers indicate the time steps shown in (**d, h**), respectively. **c, g** Corresponding applied voltage pulses. Colors follow the gate color-coding in Fig. 1. **d** First excited states for the time steps shown in (**b**), corresponding to time steps shown in Fig. 1f. Teal, green and blue sequence correspond to the 95 %, 68 %, 1 % levels of the probability density for the first excited state. **h** Ground states and first excited states for the time steps shown in (**f**). 95 %, 68 %, 1 % probability density levels of the ground states shown in yellow, orange and red, respectively. First excited states follow color map in (**d**).

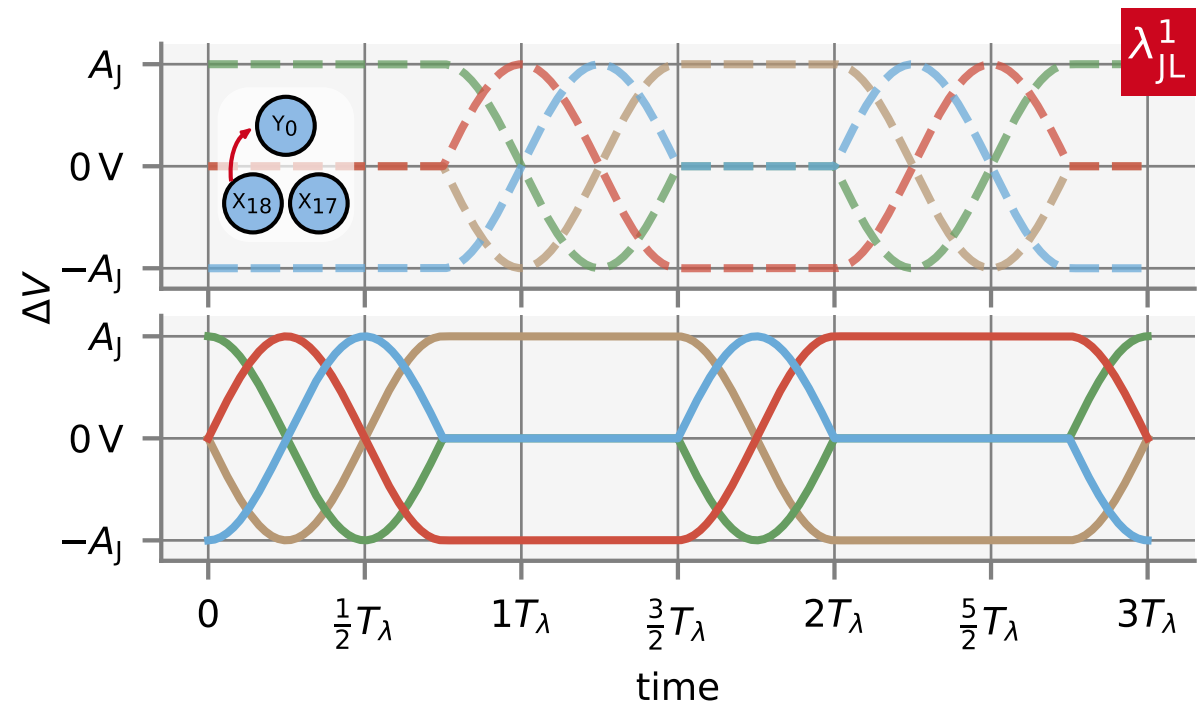

Supplementary Figure 2. **Junction transfer atomic pulse** λ^1_{JL} This figure follows the legend in Fig. 1. The atomic pulse shown shuttles the charge in QD X_{18} to QD Y_0.

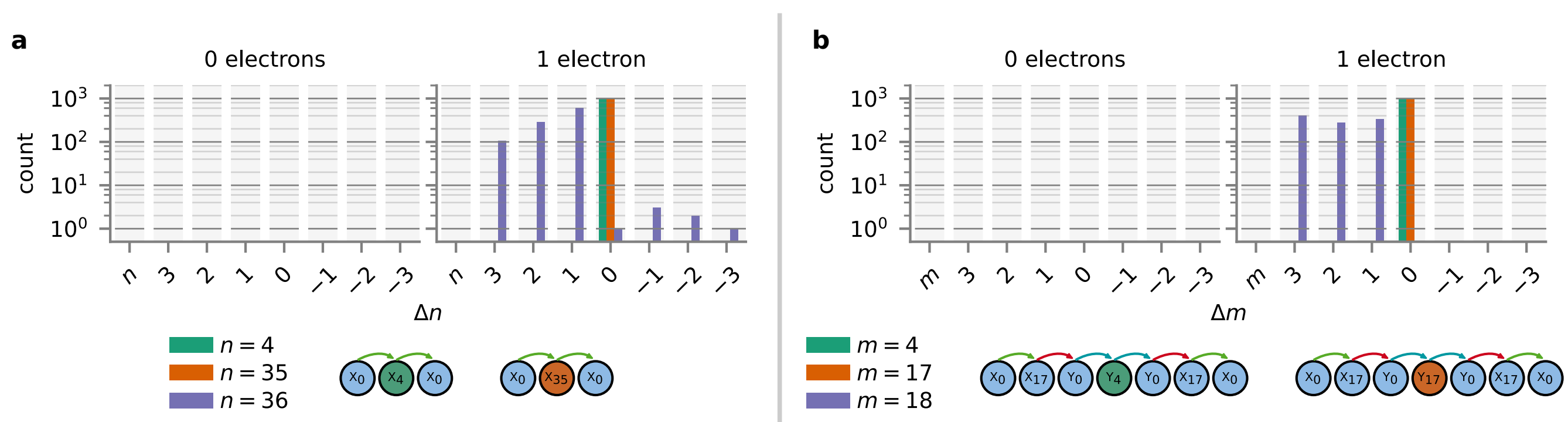

Supplementary Figure 3. **Verifying shuttling in the x- and y-shuttles.** Complementary results of Fig. 2 at $v_\lambda = v_\mathrm{J} = 28\,\mathrm{mm\,s^{-1}}$. **a** Shuttling in the x-shuttle. **b** Shuttling in the x-shuttle up to QD X_{17} followed by the y-shuttle.

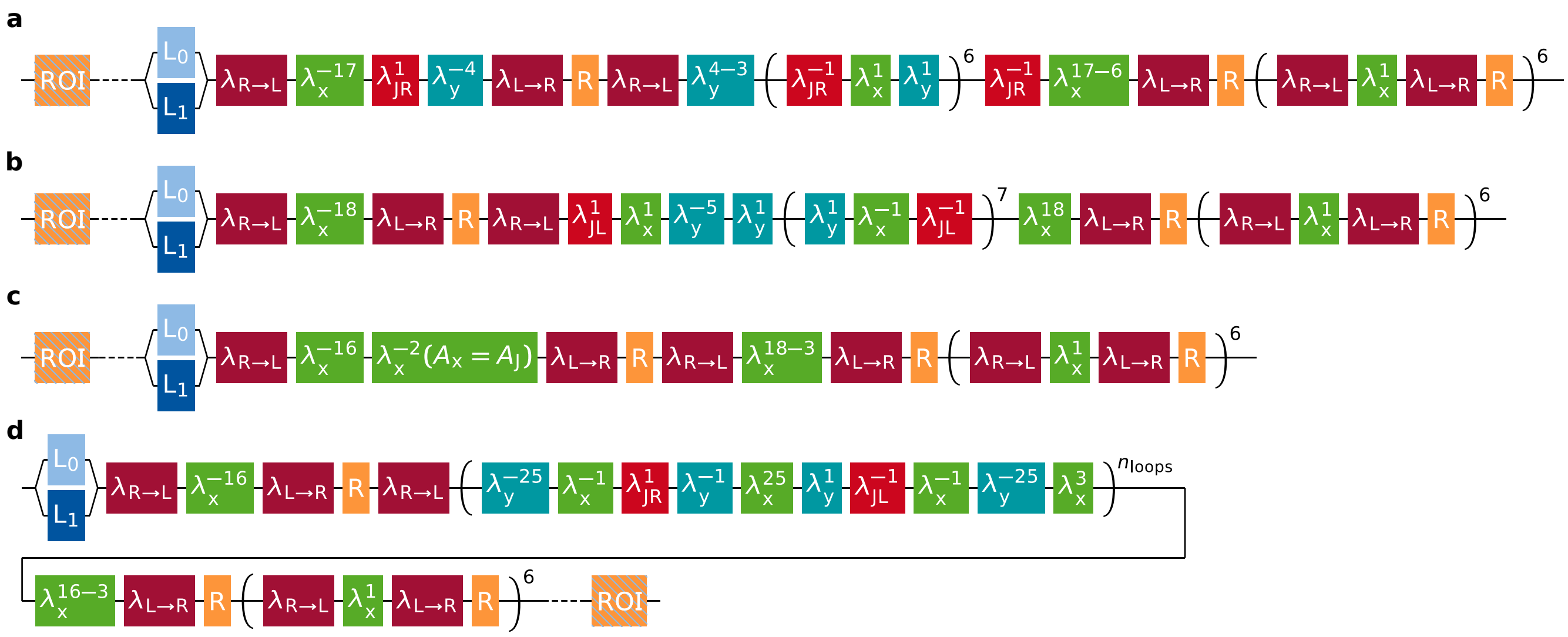

Supplementary Figure 4. **Pulses applied to explore parameter space of junction shuttling.** **a-c** Pulses used for results in Fig. 3a. **d** Pulse used for results in Fig. 3b and Fig. 3c. Note that pairs of $\lambda_{\mathrm{L}\rightarrow\mathrm{R}}\lambda_{\mathrm{R}\rightarrow\mathrm{L}}$ and their reverse are removed by a simplification step before playback.

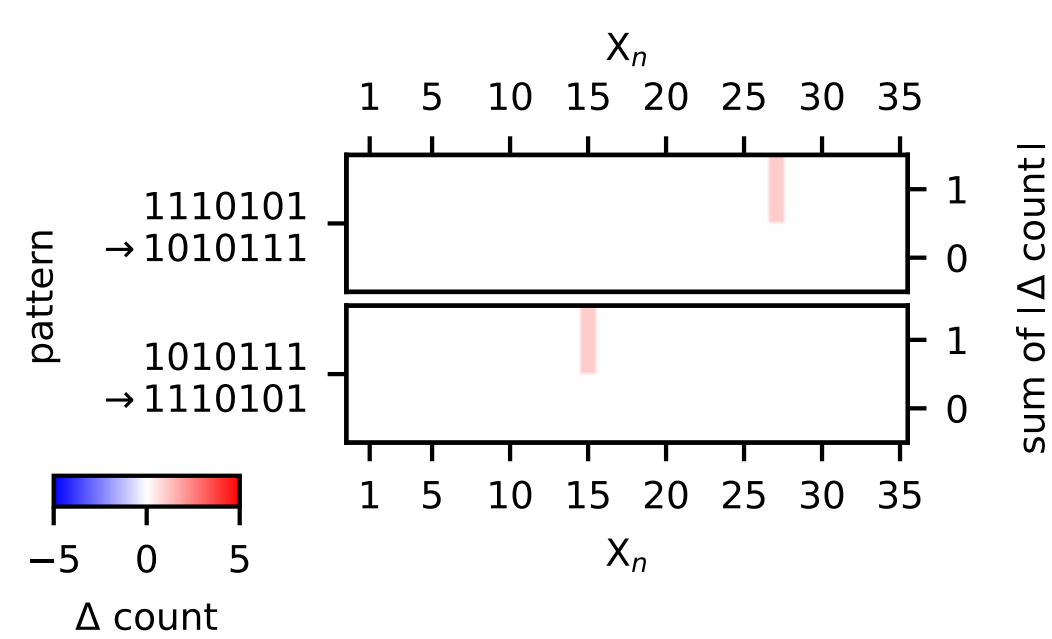

Supplementary Figure 5. **Error counts for pattern SWAP.** Complementary data for Fig. 4d following the same color map.

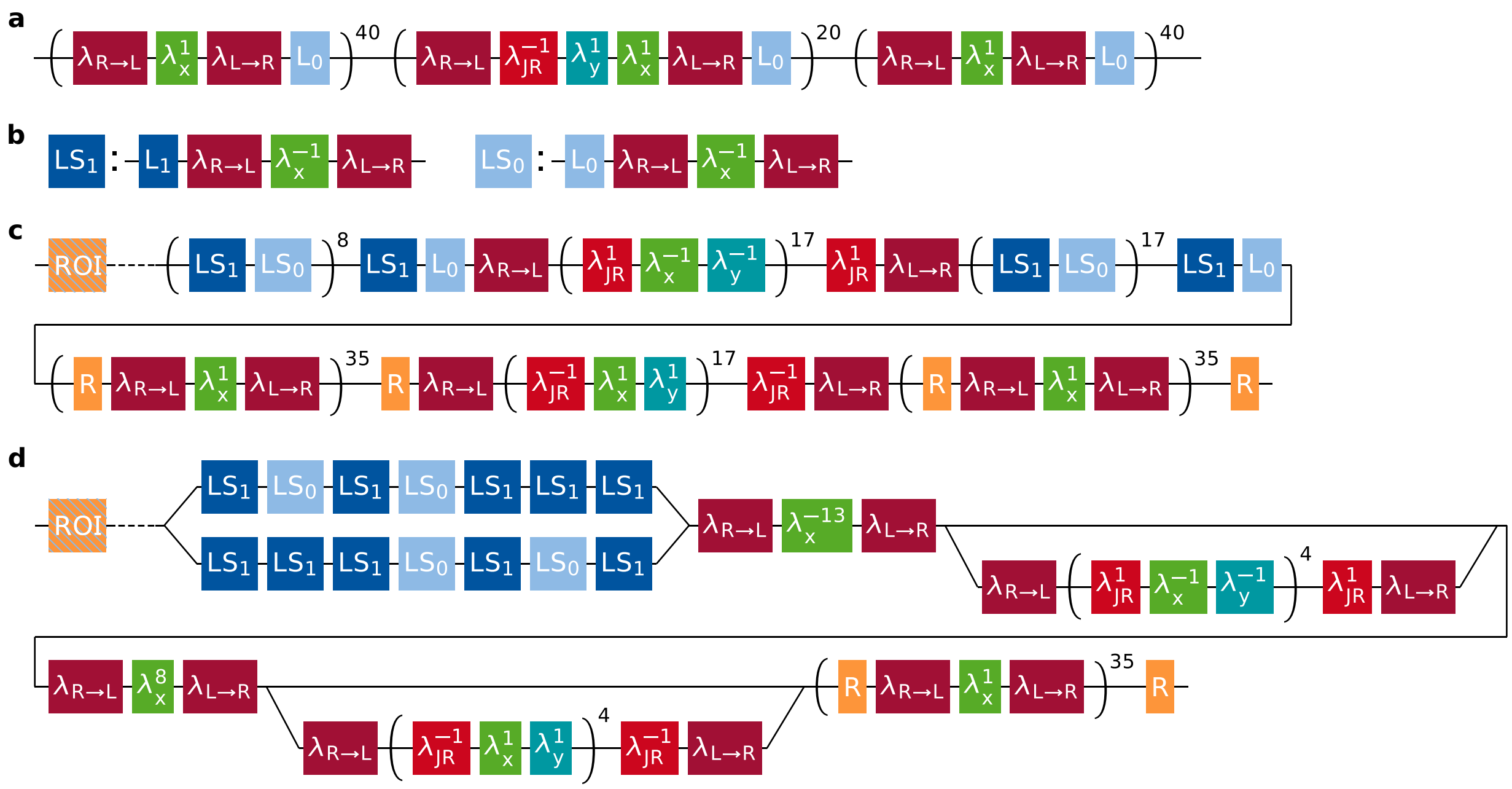

Supplementary Figure 6. **Additional pulses.** **a** Flush pulse. Due to the extensive use of slow L_0 pulses, the flush is not performed before every pulse repetition. During the flush $A_J = 260\,\mathrm{mV}$ and the same shuttle velocities used during the rest of the pulse are applied. **b** Load and shuttle notation shorthand. **c** Exemplary pulse for shuttle of $\overline{1}0$-electron pattern. For other electron patterns, only the initialization pulses are modified accordingly. **d** Pulse applied in Fig. 4c and Fig. 4d to perform SWAP of an electron pattern. The top and bottom initialization branches (first row) as well as the top and bottom shuttling branches (second row) are combined to yield 4 different pulses. The bottom shuttling branch performs the SWAP. Note that pairs of $\lambda_{\mathrm{L}\to\mathrm{R}}\lambda_{\mathrm{R}\to\mathrm{L}}$ and their reverse are removed by a simplification step before playback.

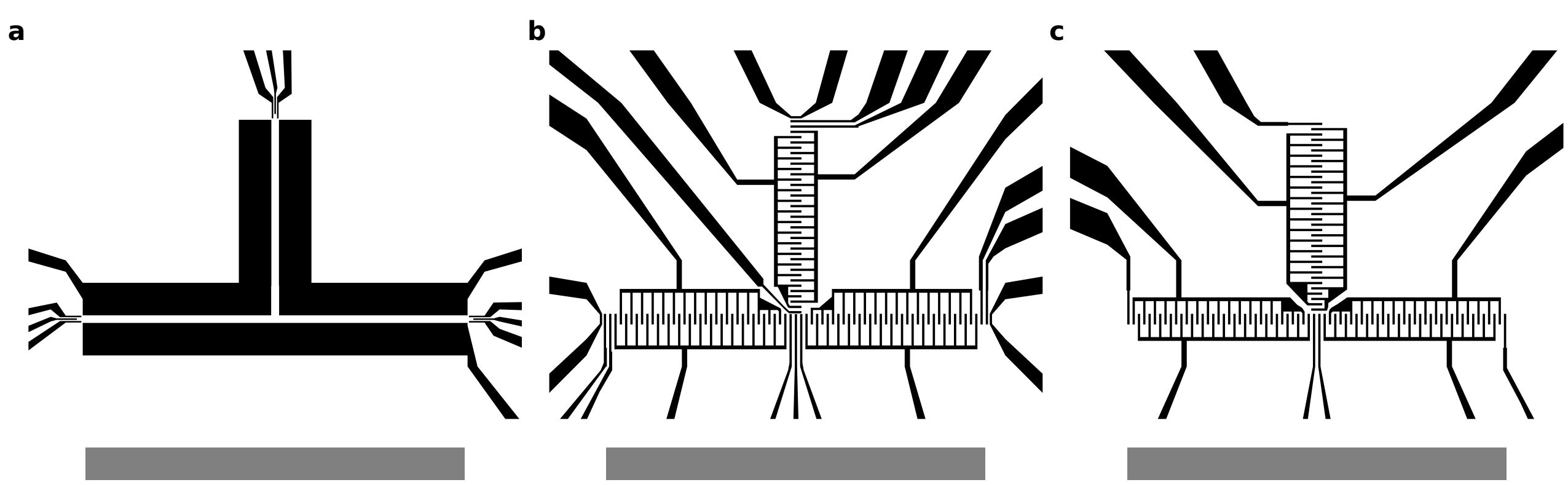

Supplementary Figure 7. **Metal gate layers defining the device.** Dark shapes correspond to metalized regions visible in Fig. 1a. Scale bars are $10\,\mathrm{\mu m}$. **a** First metal layer. **b** Second metal layer. **c** Third metal layer.

[1] Langrock, V. *et al.* Blueprint of a scalable spin qubit shuttle device for coherent mid-range qubit transfer in disordered Si/SiGe/SiO_2. *PRX Quantum* **4**, 020305 (2023).
[2] Volmer, M. *et al.* Mapping of valley splitting by conveyor-mode spin-coherent electron shuttling. *npj Quantum Inf.* **10**, 61 (2024).
[3] Struck, T. *et al.* Spin-EPR-pair separation by conveyor-mode single electron shuttling in Si/SiGe. *Nat. Commun.* **15**, 1325 (2024).
