# Supplementary material for: Conveyor-mode electron shuttling through a T-junction in Si/SiGe
Source: Nat Commun. 2026 Nov 5;17:11453. doi: 10.1038/s41467-026-78805-1 (PMC13740304; doi:10.1038/s41467-026-78805-1)
Supplement: Supplementary file 2 [file 41467_2026_78805_MOESM2_ESM.pdf]

nature portfolio

Peer Review File

Conveyor-mode electron shuttling through a T-junction in Si/SiGe

Corresponding Author: Dr Lars Schreiber

This file contains all reviewer reports in order by version, followed by all author rebuttals in order by version.

Version 0:

Reviewer comments:

Reviewer #1

(Remarks to the Author)
Beer et al. reports a T-shape junction structure for the conveyor-mode shuttling technique in silicon spin qubits. Silicon spin qubits have a weak point in the qubit connectivity as their native interaction (exchange interaction) reaches a range of just less than 100 nm. This limited connectivity imposes severe constraints on the arrangement of the spin qubits, if they are stationary. Coherent shuttling of the spin qubits is expected to lift the constraints as qubits can be arranged sparsely and transferred into the interaction range when the interaction is demanded, opening up the scalability of silicon spin qubits. While a disadvantage of shuttling is number of gate electrodes to define a shuttling lane, the conveyor-mode shuttling technique enables ones to dramatically suppress the number of gate electrodes to define a shuttling lane. The T-junction structure can joint such shuttling lanes and expand the silicon spin qubit architecture two dimensionally, highly demanded in the field of the silicon-based quantum computing.
While the importance of the T-junction, I have to say achievements in this work are moderate. First of all, spin coherent shuttling is not achieved in this work. They have demonstrated almost perfect charge shuttling through the T-junction, it does not guarantee high-fidelity spin coherent shuttling. The slow electron velocity 270 mm/s in this work implies that there are still some challenges to overcome before realizing the spin coherent shuttling through the T-junction. In an earlier work ref. 21 from the same group, charge shuttling using a conveyor-mode shuttling lane drew a lot of interest certainly. But this is because of the impactful reduction in the number of gate electrodes to implement a shuttling lane over >10 um. Given that another paper demonstrating spin coherent shuttling by the conveyor mode is published recently (ref. 19), it should be difficult for a report just about charge shuttling to draw a lot of interest nowadays. I also note that two dimensional carrier transfer over quantum-dot sites has already been demonstrated while in germanium [Floor van Riggelen-Doelman, Nature Commun. 15, 5716 (2024)]. I could not find a big technical advancement from this published work in the present work because the local structure of the T-junction is similar to such a two-dimensional quantum dot array.
Separately from these major comments, I am concerned about the presentation of this manuscript. The text contains notation fluctuations, grammatical errors, and indirect phrasing. The figures are referenced at the points where readers would typically expect them. These points make the manuscript less accessible. I recommend the authors to make substantial revisions so as to adhere to the conventions of academic writing.
All in all, I do not think that this manuscript deserves publication in Nature Communications. I recommend to transfer to more specific journals after revisions.

Reviewer #2

(Remarks to the Author)
This manuscript reports the authors' tremendous efforts in shuttling electrons through T-junction arrays of quantum dots (QDs) on a Si/SiGe platform. They demonstrate that, with a remarkably high fidelity, that they can transfer an electron from a QD array in the X direction to an array in the Y direction, and vice versa. They perform careful statistical studies to determine the electron-transfer fidelity. They also demonstrate looping operations at the T-junction, as well as shuttling of multi-electron patterns.
Overall, the quality of their experimental demonstration and analysis is excellent, and their impact on the development of semiconductor-based qubit devices is expected to be large. For these reasons, I am happy to recommend the publication of this manuscript in Nature Communications. I have a few comments on their style of presentations, which I list below. I hope that the authors could revise on them to improve the readability for general readers.
(1) The first thing that confused me was the notation of charge transfer fidelity in the abstract. I was not familiar with the

format, and I wonder how many of the readers of Nature Communications are familiar with the format. It turns out that the authors provide its definition in the METHODS section, which is very helpful, and the definition is actually very simple. But this reference is not given until Page 5, which I suspect would frustrate many readers. I suggest that the authors add a reference to the METHODS section in the Introduction paragraph where they first introduce the fidelity number.
(2) On page 1, in the first paragraph of the RESULTS section, the authors explain the position of single electron transistors (SETs) that are to be used as a charge detector. However, they are not marked in Fig. 1a. Also, if I understand correctly, there are three SETs, one each at the end of lane (top centre, bottom left, and bottom right in Fig. 1a), but only one of them (bottom right) is used in this work. This is not clearly explained in the text.
(3) In Fig. 1d, there is a blue vertical dashed line as in Fig. 1c, but there is no description in the figure caption.
(4) On Page 3, the second paragraph in the “Single electron shuttling” section, the authors describe how they tested the shuttling of an electron in the X direction. It took some time for me to understand what they did. Initially, I thought they would check the charge on the destination QD (in this case X35) for n = 35, but this is not the case. I then realised that, in order to test transfer to intermediate QDs (0 < n < 35), their method of checking reversal transfer is more effective. If the authors could more explicitly describe the reason why they chose this method in the manuscript, it would make easier for readers to understand the process.
(5) In the next paragraph, the authors mention that the distributions of electrons in \delta n = 1, 2, 3 for n = 36 are the finger print that the electron actually reached X35. I agree with this logic, however, there are also distributions in \delta n = -1, -2, -3, but I do not understand why electrons would appear there. Can authors clarify?
(6) On page 4, the authors describe transfer from X arrays to Y arrays, but use the same notation n for the number of pulses. This can be confusing (as it is not clear whether n refers to the number of X pulses, Y pulses, or the total). Can they introduce another variable for the number of Y pulses, such as m?

Version 1:

Reviewer comments:

Reviewer #1

(Remarks to the Author)
Beer et al. addressed my comments where I expressed concerns regarding the significance of their work. Most of the responses are plausible and I now have a better understanding of the importance of their work, while I have a comment about the shuttling velocity as follows:
The authors wrote "the achieved velocity 270 mm/s is already by a factor of 2000 faster than the velocity of 0.14 mm/s that we demonstrated in the linear shuttle devices published in Nature Communications". The 2000-fold improvement in the velocity is true, but I think that is just because the velocity of 0.14 mm/s in the previous publications was too slow. Rather than comparing the previous publication, if “the demonstrated shuttle velocity here is expected to be sufficient for coherent spin qubit operation across the entire device”, the authors should establish an appropriate target velocity and compare 270 mm/s with it.
All in all, the revised publication conveys the significance of the results more clearly. I am now supportive of publication in Nature Communications and will agree after the authors respond to the above comment.

Reviewer #2

(Remarks to the Author)
In this revised manuscript, the authors have made significant changes to improve the readability. I am satisfied by their response on my previous comments. As for the criticism raised by the other referee on the lack of demonstration of the spin-coherent transfer, I am in support of the authors’ claim that their focus on charge transfer fidelity is more than enough achievement. The manuscript is already rich with technical details on the technical methods of charge transfer through T junction and testing methods for the fidelity verification. In my opinion, their achievement is strong enough to justify publication in Nature Communications. Adding an aspect of spin-coherent transfer would make the paper too complicated. I therefore recommend the publication of this manuscript.

Open Access This Peer Review File is licensed under a Creative Commons Attribution 4.0 International License, which permits use, sharing, adaptation, distribution and reproduction in any medium or format, as long as you give appropriate credit to the original author(s) and the source, provide a link to the Creative Commons license, and indicate if changes were made.
In cases where reviewers are anonymous, credit should be given to 'Anonymous Referee' and the source.
The images or other third party material in this Peer Review File are included in the article's Creative Commons license, unless indicated otherwise in a credit line to the material. If material is not included in the article's Creative Commons license and your intended use is not permitted by statutory regulation or exceeds the permitted use, you will need to obtain permission directly from the copyright holder.
To view a copy of this license, visit https://creativecommons.org/licenses/by/4.0/

we would like to thank you and the reviewers for the time taken to assess our manuscript as well as for the knowledgeable review. With their help our manuscript improved considerably. With this letter, we want to lay out our point-by-point reply to the comments of the two reviewers and attach our updated manuscript. All changes are marked in red in the markup version of our manuscript. We also updated the references of preprints published in journals and added recent preprints.

In addition, we have addressed accessibility concerns by replacing red / green color pairings by magenta / green color pairings in figure 4, supplementary figure 1 and supplementary videos 1 and 2. We did not modify gate colors, including associated voltage pulses, as they are analogous to our previous publication Nat. Commun. **15**, 2296 (2024).

Response to Reviewer #1

Beer et al. reports a T-shape junction structure for the conveyor-mode shuttling technique in silicon spin qubits. Silicon spin qubits have a weak point in the qubit connectivity as their native interaction (exchange interaction) reaches a range of just less than 100 nm. This limited connectivity imposes severe constraints on the arrangement of the spin qubits, if they are stationary. Coherent shuttling of the spin qubits is expected to lift the constraints as qubits can be arranged sparsely and transferred into the interaction range when the interaction is demanded, opening up the scalability of silicon spin qubits. While a disadvantage of shuttling is number of gate electrodes to define a shuttling lane, the conveyor-mode shuttling technique enables ones to dramatically suppress the number of gate electrodes to define a shuttling lane. The T-junction structure can joint such shuttling lanes and expand the silicon spin qubit architecture two dimensionally, highly demanded in the field of the silicon-based quantum computing.

We thank the reviewer for highlighting the importance of the conveyor-mode shuttling T-junction. Indeed, the extension to two dimensionality is highly demanded for spin qubit architectures and our T-junction is a very important step in this direction**. We also agree that the suppression of the "number of gate electrodes" or more precisely the number of signal channels required for shuttling is the key to scalability of classical single electron electronics and of spin-qubit architectures**. Therefore, keeping this spirit, we focus on this suppression aspect in this work and demonstrate that our T-junction design works for single electrons with high fidelity.

(1) While the importance of the T-junction, I have to say achievements in this work are moderate. First of all, spin coherent shuttling is not achieved in this work.

The problem of spin qubit shuttling in conveyor-mode can be separated in two subproblems. **First problem is the transfer of a single charge across large distance with few signal channels and with very high fidelity.** Second problem is to preserve the spin coherence during the conveyor-mode shuttling.

The first problem addresses the **design of a device, i.e. gate pattern, layer stack, control signals in order to achieve high shuttle fidelity despite the unavoidable electrostatic disorder in the Si/SiGe quantum well.** This process requires numerical device simulations and optimization. We published the charge shuttling in a 10 µm linear shuttle device in Nature Communications two years ago (Ref. 21, new Ref. 23), which followed our simulation-based device blueprint from Ref. 26. Our conveyor-belt T-junction has never been simulated nor demonstrated.

The **spin coherent shuttling aspect has been shown in several papers** in short devices (see Ref. 18, 19, 22, new 20, 21, 24). We and others showed that it works if the charge shuttling is **working provide the valley splitting is sufficiently high**. We discussed the impact of the valley splitting on spin-qubit shuttling in our recent publication published in Nature Communications 2026 (Ref. 24, new 26). **The spin coherent shuttling is less related to suppression of the number of signal channels, but it is merely related to material aspects, i.e. large valley splitting.** Currently, this material aspect is addressed by many groups and not only in the context of spin qubit shuttling. **The valley splitting inhomogeneity of the Si/SiGe platform is a general challenge and solutions are coming up at the horizon**.

We added on page 1: "…valley splitting energy is sufficiently large [26]. The latter is subject of current research [35–39] and sufficiently large valley-splitting has been achieved across a multi quantum dot device with an optimized Si/SiGe heterostructure [2]."

Here we address the **demonstration of shuttling of a single electron with very high fidelity using few signal channels, simple control signals and a device design robust to electrostatic disorder.** Note that the existence of such a T-junction does not directly follow from the demonstration of a linear shuttle, as can be seen from the struggle of the **ion-trap qubits. For them, routing ions in junctions has been challenging and has been demonstrated rather late.**

We added a comment to the manuscript on page 1: "Shuttle junctions and qubit routing pose a challenge even for other, arguably more mature qubit platforms such as ion-qubits, for which linear shuttling has been demonstrated much earlier [32–34]"

We also want to point out that our T-junction device is not only envisioned in the context of a quantum computing with spin qubits. **It is also a significant milestone in single electron electronics and two-dimensional charge routing** with additional applications for novel classical electronics based on single electrons, i.e. low power electronics, dense memory, charge pumps for metrology.

We added to the manuscript on page 7: "This high level of single electron control might also be exploited for novel low-power classical electronics and dense memories operating with single electrons in QDs below 1 K. It might also become useful in metrology for current standards based on electron counting."

(2) They have demonstrated almost perfect charge shuttling through the T-junction, it does not guarantee high-fidelity spin coherent shuttling.

We agree that our charge shuttle infidelity across the T-junction is with 1E-7 % exceptionally small and almost perfect.
We also agree that long-range spin coherent shuttling on top of the high-fidelity charge shuttling requires more progress**. But this is a material related progress, mainly valley-splitting and isotopically purification of Si and Ge in order to reduce hyperfine interaction.** While the isotopically purification is merely a problem of funding and logistics, increasing the valley splitting is an important material aspect for the Si/SiGe platform and a very timely topic covered by many publications recently with upcoming solutions at the horizon. We discussed the impact of valley splitting in Nature communications (Ref. 24, new 26).

We added to the manuscript on page 1: "…and the valley splitting energy is sufficiently large [26]."

But this material aspect is not our focus in the manuscript here. **We focus on the design of the T-junction and demonstrate nearly perfect charge shuttling with minimal number of signal channels. More precisely, we demonstrate an infidelity of charge shuttling well below the values reported in any other publication by multiple orders of magnitude.** Detection of such very low infidelity has been substantially aided by looping within 2-dimensional device topology, which renders the detection of otherwise self-compensating errors possible. The charge shuttling fidelity serves as the absolute lower bound of the fundamental operation of shuttling-based architectures the concern for which is finally eliminated with this work. Finally, we want to directly respond to the remark of the reviewer that a solution of the valley splitting problem in a dense quantum dot matrix in turn does not guarantee a two-

dimensional quantum computing architecture either. The T-junction is a separate achievement.

We added to conclusion of the discussion on page 7: "In short lanes, spin-qubit shuttling with high velocity [21] and spin manipulation [44] has been demonstrated. While it is significant to design and demonstrate devices for conveyor-mode charge routing with minimal control complexity, note that spin-qubit shuttling in Si/SiGe requires material progress mainly on homogeneous enhancement of valley-splitting across large areas of the devices [26]."

(3) The slow electron velocity 270 mm/s in this work implies that there are still some challenges to overcome before realizing the spin coherent shuttling through the T-junction.

The electron shuttling velocity in this work **was only limited by the signal lines** installed in the cryostat, which had a bandwidth of ~1 MHz and we hit this bandwidth limit. Admittedly, this is not ideal as we cannot show the full potential of our T-junction design. Importantly, we found no evidence that the T-junction limited the velocity. Note also that **the achieved velocity 270 mm/s is already by a factor of 2000 faster than the velocity of 0.14 mm/s that we demonstrated in the linear shuttle devices published in Nature Communications** (Ref. 20, new 22).
More importantly, the demonstrated **shuttle velocity here is expected to be sufficient for coherent spin qubit operation across the entire device** depending on remaining hyperfine interaction and optimization of valley splitting. There is no indication from our work that device operation was limited by device geometry. Faster linear conveyor-mode shuttling operation, albeit across much shorter sub 1 µm distances have already been achieved in other works (Ref. 19, new 21 and Ref. 22, new 24).

We added to the manuscript on page 7: "…and the shuttle velocity of 270 mm s^{-1} is 2000 times higher than achieved in the longest linear shuttle device demonstrated so far [23]…"

(4) In an earlier work ref. 21 from the same group, charge shuttling using a conveyor-mode shuttling lane drew a lot of interest certainly. But this is because of the impactful reduction in the number of gate electrodes to implement a shuttling lane over >10 um. Given that another paper demonstrating spin coherent shuttling by the conveyor mode is published recently (ref. 19), it should be difficult for a report just about charge shuttling to draw a lot of interest nowadays.

Yes, our demonstration of **linear charge shuttling in conveyor-mode drew a lot of interest** and got published in in Nature Communications. Spin qubit shuttling in short devices has been

shown by us and others and will likely work in longer devices, if material issues, mainly the valley splitting, are solved.

Therefore, the really important step here is routing in a T-junction with few signal channels and simple signals. It paves the way to two-dimensional architectures with single electrons or spin qubits. We want to point out that **the number signal channels and type of signals channels of a T-junction is an important input parameter for circuit designer of cryo-electronics.** Our T-junction design and control drew a lot of interest on many conferences (SiQEW 2025, APS March meeting 2026, Spin Qubit 7, ICPS, and many more) and got highlighted as a major progress in several project reports.

We added to the text on page 7: "The number and simplicity of signal channels is crucial for the co-integration of cryo-electronics [41], which in turn is a prerequisite for scalable architectures [42]. Our work might guide these circuit designs [43]."

Without doubt compared to Ref. 21, new 23, a **significant increase in the charge routing complexity without a substantial increase in control complexity has been achieved by us here.** We substantially exceed the shuttled distance, both in the spatial extend described by the path as well as the cumulated distance traversed by a single electron (exceeding 10^6 * 10 * 280 nm ~ 2.8 m, as can be derived from Table 1).

(5) I also note that two dimensional carrier transfer over quantum-dot sites has already been demonstrated while in germanium [Floor van Riggelen-Doelman, Nature Commun. 15, 5716 (2024)]. I could not find a big technical advancement from this published work in the present work because the local structure of the T-junction is similar to such a two-dimensional quantum dot array.

On this point, we disagree with the opinion of the reviewer. The local structure of the T-junction significantly differs from the simple 2x2 quantum dot array presented in van Riggelen-Doelman et al.. Due to the fundamental nature of conveyor mode shuttling enabling continuous translation of the confining quantum dot potential, the T-junction device does not feature distinct barrier and plunger gates in contrast to van Riggelen-Doelman et al.. On the one hand, this makes the required T-junction transfer significantly more difficult as the symmetry of the achieved confinement potential is broken (see e.g. Sup. Fig. 1.) **On the other hand, our T-junction renders sparse qubit architectures with co-integration of cryoelectronic possible (e.g. Ref. 13, new 15 (Spider-web array), Ref. 27, new 29 (SpinBus architecture)), in contrast to simpler dense quantum dot arrays.**

Nevertheless, there is certainly potential in the dense arrays and therefore we mention dense qubit array in the introduction, also refer to Riggelen-Doelman et al. and other related publications.

(6) Separately from these major comments, I am concerned about the presentation of this manuscript. The text contains notation fluctuations, grammatical errors, and indirect phrasing. The figures are referenced at the points where readers would typically expect them. These points make the manuscript less accessible. I recommend the authors to make substantial revisions so as to adhere to the conventions of academic writing.

We agree with the reviewer's comment and regret the formal state of the submission. Therefore, we made significant changes to phrasing and notation also addressing feedback by Reviewer 2. We also let an English native speaker proof read the article (see many small changes in the markup version of the manuscript).

Response to Reviewer #2

This manuscript reports the authors' tremendous efforts in shuttling electrons through T-junction arrays of quantum dots (QDs) on a Si/SiGe platform. They demonstrate that, with a remarkably high fidelity, that they can transfer an electron from a QD array in the X direction to an array in the Y direction, and vice versa. They perform careful statistical studies to determine the electron-transfer fidelity. They also demonstrate looping operations at the T-junction, as well as shuttling of multi-electron patterns. Overall, the quality of their experimental demonstration and analysis is excellent, and their impact on the development of semiconductor-based qubit devices is expected to be large. For these reasons, I am happy to recommend the publication of this manuscript in Nature Communications.

We would like to express our gratitude to the reviewer for they thorough analysis and **the clear recommendation of publication of our work in Nature Communications.**

I have a few comments on their style of presentations, which I list below. I hope that the authors could revise on them to improve the readability for general readers.

We thank the reviewer for their comment. We think it improved the readability of the manuscript a lot.

(1) The first thing that confused me was the notation of charge transfer fidelity in the abstract. I was not familiar with the format, and I wonder how many of the readers of Nature Communications are familiar with the format. It turns out that the authors provide its definition in the METHODS section, which is very helpful, and the definition is actually very simple. But this reference is not given until Page 5, which I suspect would frustrate many readers. I suggest that the authors add a reference to the METHODS section in the Introduction paragraph where they first introduce the fidelity number.

We acknowledge that the presentation of the two-sided uncertainty of a determined success rate (Fidelity) is not fully established in the community. We identified this as common weakness in the typical statistical analysis of high success rates in the community, leading to nonsensical results such as usually derived confidence intervals exceeding 100 %.

We have added a reference to the subsection in the Methods section as requested: "(See methods section on notation)" on page 1.

(2) On page 1, in the first paragraph of the RESULTS section, the authors explain the position of single electron transistors (SETs) that are to be used as a charge detector. However, they are not marked in Fig. 1a. Also, if I understand correctly, there are three SETs, one each at the end of lane (top centre, bottom left, and bottom right in Fig. 1a), but only one of them (bottom right) is used in this work. This is not clearly explained in the text.

Indeed, there are three SETs available in principle. As requested, we have modified Fig. 1a to mark the used SET by yellow gates and added a short explanation in the caption of Fig. 1: "Gates defining the sensor used for electron charge readout are shaded yellow." We also modified panel Fig. 1a to indicate gates noted in Methods that were not labelled in the figure before.

As demonstrated the closed barriers present at the left end of the x-shuttle as well as the top end of the y-shuttle allow us to detect full transfer of the shuttled electron across the entire device by detecting its untimely return at the right SET. The use of additional SETs would not have provided any additional evidence to justify the increased complexity of the experiment due to required tuning. In addition, the same technique was employed in Ref. [21] which allows the reader to directly compare the results without the required justification of a new technique.

We added an explanation to the text on page 3: "In order to keep the number of signal channels as low as possible to simplify control, we only use the right SET in this work."

(3) In Fig. 1d, there is a blue vertical dashed line as in Fig. 1c, but there is no description in the figure caption.

We have identified additional issues with the caption of Fig. 1 and deeply regret any confusion we might have caused.

We modified the caption of Fig. 1, accordingly: "**d**… Simulated electron wave function for the voltage configuration indicated by the red dashed line is shown in panel f**…"**

"f Simulated potentials φ and electron ground state probability densities (yellow-red circular shapes) at three positions in the device corresponding to three points in time. Claviature gates are shown as outlines and screening gates are indicated as a light gray grid. Blue arrows: Electron states in \qdn{X}{16} and \qdn{Y}{0} in the idle state. Red arrow: Electron during inter-lane junction transfer)…"

We also changed the color of the dashed line in Fig. 1d to correspond to the associated arrow in Fig. 1f.

(4) On Page 3, the second paragraph in the "Single electron shuttling" section, the authors describe how they tested the shuttling of an electron in the X direction. It took some time for me to understand what they did. Initially, I thought they would check the charge on the destination QD (in this case X35) for n = 35, but this is not the case. I then realised that, in order to test transfer to intermediate QDs (0 < n < 35), their method of checking reversal transfer is more effective. If the authors could more explicitly describe the reason why they chose this method in the manuscript, it would make easier for readers to understand the process.

We have modified the second paragraph of the section to clarify the experimental procedure. We added e.g. on page 4 "To check whether the electron can reach the end of the x-shuttle, we probe the effect of shuttling beyond the end of the x-shuttle similar to Ref. [23]."

(5) In the next paragraph, the authors mention that the distributions of electrons in \delta n = 1, 2, 3 for n = 36 are the finger print that the electron actually reached X35. I agree with this

logic, however, there are also distributions in \delta n = -1, -2, -3, but I do not understand why electrons would appear there. Can authors clarify?

We thank the reviewer for his careful reading of all aspects of our work. Our not fully conclusive explanation is that the shuttled electron is shuttled/tunneled into the electrostatically disordered barrier at the end of the device. In few cases in might only tunnel out by repeated λ^{-1}_x pulses. This might cause non-deterministic delays in few case and thus clicks at \delta n = -1, -2, -3.

We have added an explanation on page 4: "In few cases, but only for n = 36, electrons are detected later than expected ($\Delta n \in \{-1, -2, -3\}$). While electrons would be delayed if λ^1_x pulses fail, the detection of these events only when the electrons are expected to reach the end of the x-shuttle implies a different mechanism. We attribute this observation to electrons shuttled or tunneled into the disordered potential barrier at the end of the shuttle being freed by repeated application of λ^1_x pulses."

We want to mention that in a previous iteration of this experiment, the shuttle channels were accidentally flooded due to larger gate voltage applied in conjunction with an accidental offset between the chemical potential of ohmic contacts of different SETs. This caused a large number of electrons to be deposited into the x- and y-shuttle which could be extracted one at a time by repeated flushing pulses. Even after multiple days of applying these pulses back-to-back, electrons were still recovered from the shuttles.

(6) On page 4, the authors describe transfer from X arrays to Y arrays, but use the same notation n for the number of pulses. This can be confusing (as it is not clear whether n refers to the number of X pulses, Y pulses, or the total). Can they introduce another variable for the number of Y pulses, such as m?

The notation was chosen to be exact and consistent even for the more complex, arbitrary pulses. However, we agree that this decreases the accessibility of the submission.

Thus, we have modified the notation as suggested by the reviewer at several positions, e.g. on page 5: "…We substitute the Δn notation to identify the nominal position of the electron with its analogue Δm for this shuttle path…"
