# Supplementary material for: Conveyor-mode electron shuttling through a T-junction in Si/SiGe
Source: Nat Commun. 2026 Nov 5;17:11453. doi: 10.1038/s41467-026-78805-1 (PMC13740304; doi:10.1038/s41467-026-78805-1)
Supplement: Supplementary file 3 [file 41467_2026_78805_MOESM3_ESM.pdf]

Description of Additional Supplementary Files

File Name: Supplementary Movie 1
Description: 'Simulated electron wavefunction, wavefunction projections and orbital splitting when shuttling an electron between the xand y-shuttle corresponding to Supplementary Figure 1a-d with A = 260 mV.'

File Name: Supplementary Movie 2
Description: 'Simulated electron wavefunction, wavefunction projections and orbital splitting when shuttling an electron between the xand y-shuttle corresponding to Supplementary Figure 1e-h with A = 100 mV.'
